# Experimentally validated simulation of coronary stents considering different dogboning ratios and asymmetric stent positioning

**DOI:** 10.1371/journal.pone.0224026

**Published:** 2019-10-18

**Authors:** Lisa Wiesent, Ulrich Schultheiß, Christof Schmid, Thomas Schratzenstaller, Aida Nonn

**Affiliations:** 1 Computational Mechanics and Materials Lab, Ostbayerische Technische Hochschule (OTH) Regensburg, Regensburg, Germany; 2 Regensburg Center of Biomedical Engineering (RCBE), Regensburg, Germany; 3 Medical Device Lab, OTH Regensburg, Regensburg, Germany; 4 Material Science and Surface Analytics Lab, OTH Regensburg, Regensburg, Germany; 5 University Hospital Regensburg, Cardiothoracic and Cardiovascular Surgery, Regensburg, Germany; University of Nottingham, UNITED KINGDOM

## Abstract

In-stent restenosis remains a major problem of arteriosclerosis treatment by stenting. Expansion-optimized stents could reduce this problem. With numerical simulations, stent designs/ expansion behaviours can be effectively analyzed. For reasons of efficiency, simplified models of balloon-expandable stents are often used, but their accuracy must be challenged due to insufficient experimental validation. In this work, a realistic stent life-cycle simulation has been performed including balloon folding, stent crimping and free expansion of the balloon-stent-system. The successful simulation and validation of two stent designs with homogenous and heterogeneous stent stiffness and an asymmetrically positioned stent on the balloon catheter confirm the universal applicability of the simulation approach. Dogboning ratio, as well as the final dimensions of the folded balloon, the crimped and expanded stent, correspond well to the experimental dimensions with only slight deviations. In contrast to the detailed stent life-cycle simulation, a displacement-controlled simulation can not predict the transient stent expansion, but is suitable to reproduce the final expanded stent shape and the associated stress states. The detailed stent life-cycle simulation is thus essential for stent expansion analysis/optimization, whereas for reasons of computational efficiency, the displacement-controlled approach can be considered in the context of pure stress analysis.

## Introduction

Ischemic heart disease is the most common cause of death world wide [1]. It is caused by arteriosclerosis, the accumulation of plaque at the inner arterial wall. Arteriosclerosis is mainly treated by percutaneous coronary angioplasty with stent placement. Thereby, a stent crimped onto a balloon catheter is minimal-invasively implanted at the narrowed part of the artery (stenosis) to restore arterial lumen and to keep the vessel open. However, vascular injuries during stenting might lead to acute inflammatory reactions of the arterial wall with subsequent neointimal hyperplasia [2] and the re-narrowing of the artery (in-stent restenosis (ISR)). Although stenting has improved over the last decades, ISR still represents a major problem occurring in about 20% of the cases during bare metal stent (BMS) implantation [3]. Drug-eluting stents (DES) coated with a special anti-proliferative layer to prevent ISR, have emerged in recent years and are an alternative to BMS. Initial clinical outcomes of DES are very promising and have shown that DES can potentially reduce ISR rate to less than 10% [4–6]. DES, however, show lower cost-effectiveness compared to BMS combined with a residual ISR risk potentially due to mechanical vascular wall injuries during stent implantation [4]. Therefore, the improvement of coatings as well as stent deployment strategies and stent designs of BMS and DES are of great importance [7, 8].

**Fig 1:**
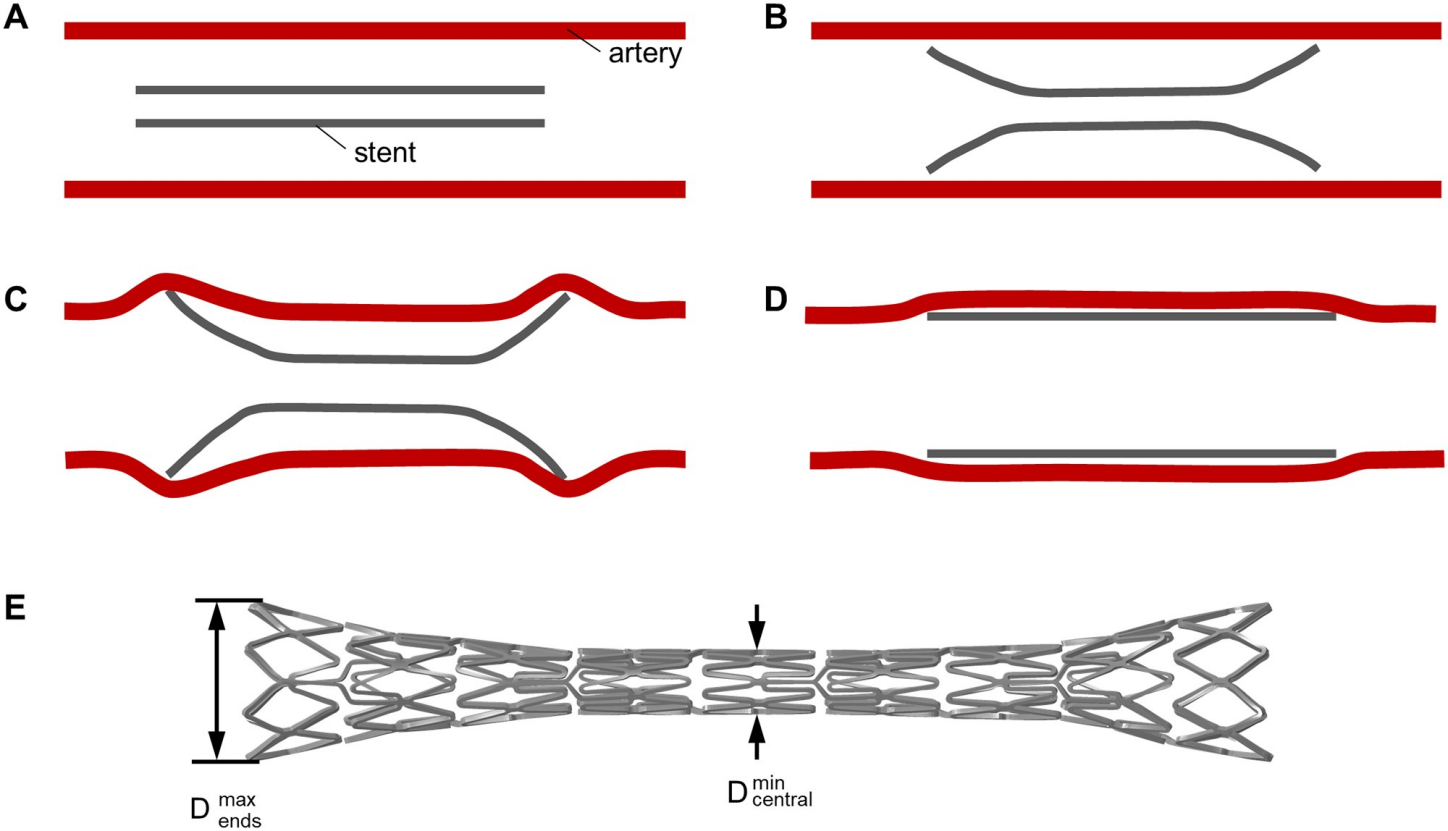
Schematic illustration of the dogboning ratio during stent deployment. A: Crimped stent inside an artery. B: Stent expansion with pronounced dogboning ratio. C: Overstretching of the artery due to the dogboning ratio. D: Final cylindrical expanded stent configuration inside slightly overstretched artery. E: Exemplary detailed illustration of the determination of dogboning ratio.

Vascular wall injuries are mainly related to expansion characteristics of stents, including the so-called dogboning (DB) effect/ratio (Fig 1). Thereby, stent expansion is pronounced at the stent ends, resulting in increased stresses and local injures at the vessel wall. DB ratio is defined as

$$\begin{array}{c} DB=\frac{D_{max,end}-D_{min,central}}{D_{max,end}}\cdot 100\% \end{array}$$ (1)

with *D_max,end_* the maximum stent diameter at the proximal/ distal stent end and *D_min,central_* the minimum stent diameter at the central part during the development of the DB effect, respectively (Fig 1).

To reduce the risk of vascular injuries and thus the incidence of ISR, the development of an expansion-optimized stent design is desired aiming at the minimization of the DB ratio.

Experimental investigations of the relation between stent design and expansion behavior are costly and difficult to perform. Numerical methods offer a valid way of efficiently determining the influence of even small design changes on stent expansion. There are numerous numerical investigations on stent expansion in literature [8–22]. These models vary in their complexity and reflect reality more or less accurately. However, these numerical analyses are commonly limited to the visual prediction of stent expansion focusing on the possibility of depicting the DB ratio without assessing its actual impact/extent. Experimental validation is hardly considered, e.g. with regard to the exact manifestation of DB ratio, stent foreshortening and recoil behavior. In order to develop an expansion-optimized stent design, however, the validation of the numerical models is necessary to provide correct boundary conditions for any optimization attempt. In the following, a brief overview of the current state of the art of stent simulation and its limitations is presented.

The first finite element analyses (FEAs) of stent expansion were performed without the consideration of the balloon [8, 12, 23–25]. These approaches were based on the assumption that the stiffness of the balloon is very low compared to the stent and is therefore negligible [9]. The stent was either load-controlled expanded by directly applying an increasing pressure onto the inner surface of the stent [10–12, 22], or displacement-controlled expanded by using a rigid/deformable cylinder as an expander [13–15]. However, these approaches could only represent an unrealistic cylindrical or a spindle-shaped stent expansion [8, 22].

Subsequent stent simulations involved the consideration of a simplified balloon configuration using an elastic/hyper-elastic cylinder without considering the semi/non-compliant material behavior of the angioplasty balloon [16–19]. In this way, the DB effect could be partially displayed for the first time. By comparing the simulation and experimental results in terms of pressure-diameter response, Kiousis et al. [18] showed that only the initial expansion of the proximal and distal stent ends could be well reproduced with this approach whereas the central diameter of the stent did not reflect reality. Wang et al. [19] further demonstrated that the stent’s foreshortening behavior obtained with this approach did not match experimental data.

A later study by de Beule et al. [8] indicated that a realistic stent expansion analysis is only feasible by considering the balloon folding pattern. This approach represents the most detailed stent model attempt to date. Thereby, the balloon folding pattern is either idealized or reconstructed on the basis of CT data. Even though there exist several stent analyses using this approach [8, 20, 21], none included a detailed validation. De Beule et al. [8] performed a brief quantitative validation based on the manufacturer compliance chart representing the relation between central stent diameter and inflating pressure. Although, the expansion behavior of the central part of the stent was validated, no statement could be made about the expansion behavior at the stent ends. The stent’s expansion pattern has been further visually compared with experimental data of a similar stent obtained from literature [22] allowing for a qualitative conformation of the DB effect but without any quantitative assessment.

The most promising stent simulation so far was performed by Bukala et al. [9] including stent crimping and stent-balloon expansion using an implicit solution scheme. However, only a partial validation was performed. For example, the crimping radial force was compared with experimental data which only matched well in the initial phase, whereas lower radial force occurred in the further course of the crimping simulation. Further, stent foreshortening and elastic recoil were compared with experimental data from similar stents in literature [26]. No detailed validation regarding the DB effect development was shown. The deviation between numerical and experimental results were attributed to the approximation of the stent geometry and the exact material parameters, which are influenced by production factors.

Based on the limitations presented in [9], it can be concluded that the influence of manufacturing parameters, including laser cutting and electropolishing, on the final stent geometry should be considered within a realistic stent analysis. In addition, material parameters based on the stent and balloon raw materials should be implemented into the numerical model. So far the balloon is commonly approximated with an elastic material [8, 9] which can only represent the semi/non-compliance of the balloon to a limited extent.

Most FEA focus on stent expansion neglecting the crimping process. Crimping induces severe residual stresses in the stent and has a decisive influence on the subsequent stent expansion, especially with regard to elastic recoil [15, 21]. Therefore, the crimping process with an appropriate material model should be included in a realistic stent FEA.

Summarizing, there are already some approaches for stent simulation using FEA. A major limitation of these stent simulations, however, is the lack or insufficient validation of the simulation approaches. Literature research performed by the authors suggests that no detailed experimentally validated numerical stent analysis is available so far.

The novelty of this research is thus the provision of an experimentally validated numerical simulation approach which is capable of realistically representing the stent life-cycle including balloon folding, stent crimping and the free expansion of the balloon-stent-system. The broad applicability of the stent life-cycle simulation approach is verified by the successful simulation and validation of two different stent designs with homogenous (V1) and heterogenous (V2) stent stiffness, as well as the simulation of an asymmetrically positioned stent V1 on the balloon catheter. To demonstrate the advantages and necessity of stent life-cycle simulations, a comparison with the commonly used displacement-controlled expansion approach was performed.

## Materials and methods

A modeling approach for the detailed simulation of the free stent expansion is presented and compared with a more standard displacement-controlled (DC) expansion approach. To demonstrate the wide applicability of the detailed simulation approach, a sensitivity analysis is performed for two stent designs and an asymmetrically positioned stent on the balloon catheter. In total four stent simulations were carried out: detailed simulation of the life-cycle of i) a stent with homogenous stiffness (V1), ii) a stent with heterogenous stiffness (V2), iii) an asymmetrically positioned stent V1 on the balloon catheter and iv) a DC expansion of the previous crimped stent with homogenous stiffness (V1). The numerical methods of the simulations are described below. As the novelty of the present work consists in a comprehensive experimental validation of the numerical results, a short overview of the experimental methods is further given.

### Detailed simulation of the stent life-cycle

Stent analysis is characterized by complex non-linearities due to high deformation, contact problems and non-linear material behavior. Therefore, the explicit solution scheme was chosen using the Abaqus/Explicit solver (Dassault Systèmes, Vélizy-Villacoublay, France). As inertia is assumed to have a negligible effect on balloon folding and stent expansion in clinical practice [8], a quasi-static approach was used within Abaqus/Explicit, which is also common practice in stent simulation [8, 17, 20, 27]. Inertial effects can be neglected if the ratio of kinetic to internal energy does not exceed a critical value of 5—10% throughout most of the process [28]. FEA of the stent life-cycle consists of several simulation steps including balloon folding, balloon pleating, stent crimping, post-crimp recoil, stent expansion and post-expansion recoil which are combined within two successive simulations: i) balloon folding and ii) stent crimping and expansion simulation.

#### Balloon folding simulation

In previous studies, balloon folding has not been explicitly reproduced by numerical simulation. Instead, the sketch of a folded balloon cross-section has been extruded and used for simulation. In this model, the balloon folding process is implemented and the folded balloon configuration subsequently passed to the final stent expansion model.

A semi-compliant Baroonda angioplasty balloon (BMT Bavaria Medizin Technologie GmbH, Germany) has been used for the balloon simulation. The cylindrical part of the balloon has length *L_bal,cyl_* of 19.86 mm, a nominal diameter *D_bal_* of 3.5 mm at a balloon pressure of 10 bar (1 MPa) and a balloon thickness *t_bal_* of 0.025 mm. The total balloon length *L_bal,tot_* is 26.6 mm. The cylindrical catheter shaft is modeled with a length *L_cat_* of 27 mm and a diameter *D_cat_* of 0.6 mm.

**Fig 2:**
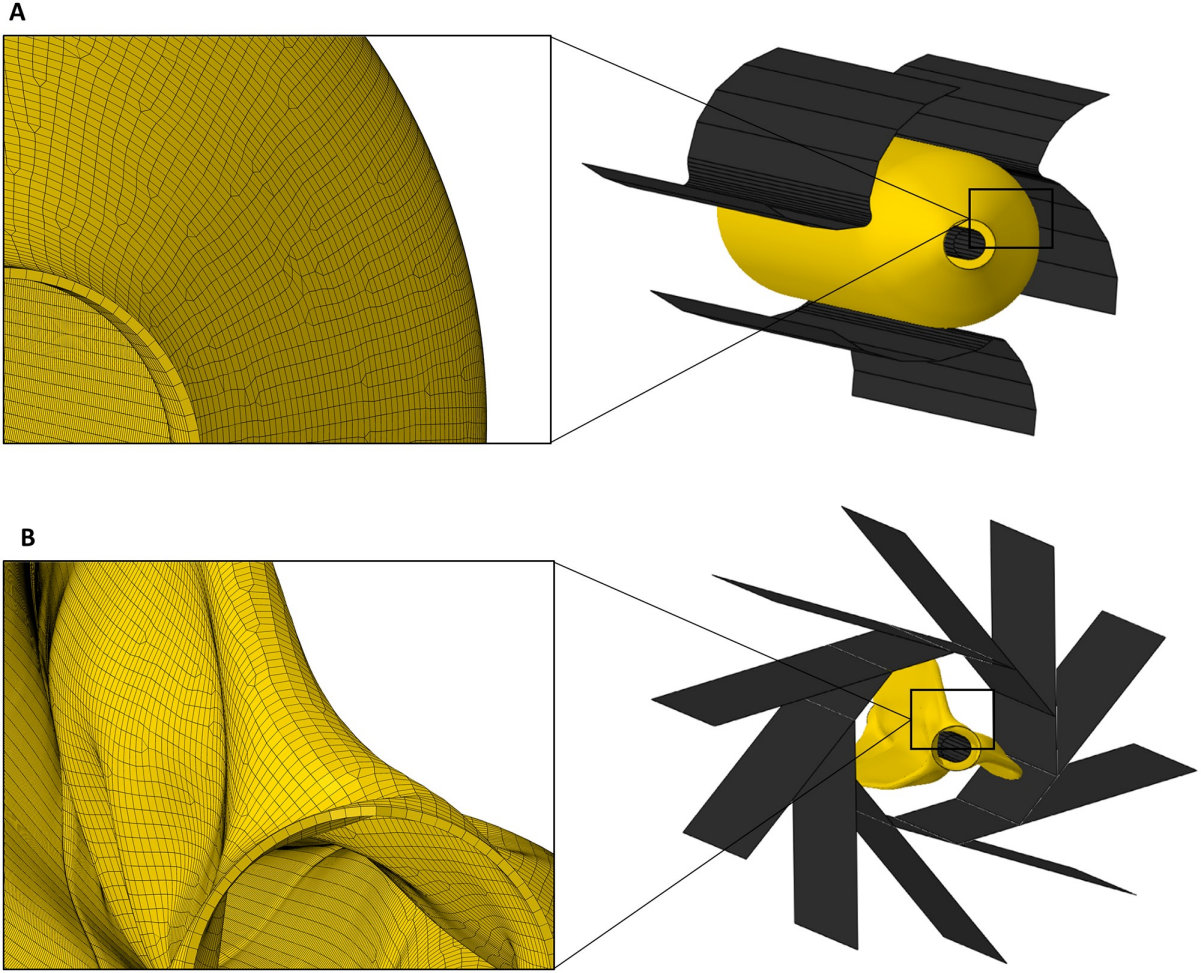
Model set-up of the balloon folding process. A: Isometric illustration of the initial balloon inside the folding jaws with the representation of the balloon mesh. B: Isometric illustration of the folded balloon inside the pleating jaws and the resulting balloon mesh after folding.

The balloon folding process consists of two steps: i) the generation of a star-shaped configuration by rotation of three folding jaws around their pivot point and ii) the wrapping of the generated folds around the catheter by rotating ten pleating jaws. The balloon folder (MSI Machine Solution Flagstaff, US) was represented by the contact surfaces of the three folding and ten pleating jaws. The pivot points of the folding jaws are located on a circle with a radius *r* of 24.13 mm and the pleating jaws on a circle with a radius *r* of 84.6 mm to the centre of the folder. Catheter, folding and pleating jaws have been implemented as rigid bodies due to their high stiffness compared to balloon material. For balloon folding, angioplasty balloon and catheter were inserted coaxially at the center of the radially arranged folding and pleating jaws (Fig 2).

The balloon consists of the polyamid Grilamid L25. In previous studies [8, 9, 25], the balloon material was implemented as an elastic material only considering the semi-compliance of the polyamid to a certain extent. In this work, the inelastic constitutive response has been described by a von Mises plasticity material model with isotropic hardening based on a uniaxial tensile test provided by the balloon manufacturer. The von Mises flow can be described as follows:

$$\begin{array}{c} \frac{1}{2}\sigma_{ij}\sigma_{ij}-\frac{1}{3}\sigma_{f}^{2}\left(\epsilon_{pl}\right)=0 \end{array}$$ (2)

with the deviatoric stress components *σ*_ij_, the flow stress *σ*_f_ which increase with plastic deformation and the equivalent plastic strain *ϵ*_pl_. After reaching the tensile strength strain hardening was considered by Hollomon’s power law equation.

$$\begin{array}{c} \sigma =K\epsilon_{pl}^{n} \end{array}$$ (3)

The strength coefficient *K* and the strain hardening exponent *n* were derived from the true stress-strain curve in the plastic region. The material parameters used for modeling the consititutive material behavior of the balloon (Grilamid L25) are summarized in Table 1.

**Table 1:** Material parameters used for modeling the constitutive material behavior of the balloon (Grilamid L25).

Density *ρ*	Young’s modulus *E*	Yield strength R_p02_	Tensile strength R_m_	Poisson ratio *ν*	Strength coefficient *K*	Strain hardening parameter *n*
1100 kg/m^3^	1000 MPa	30 MPa	40 MPa	0.1	61.2 MPa	0.12

**Fig 3:**
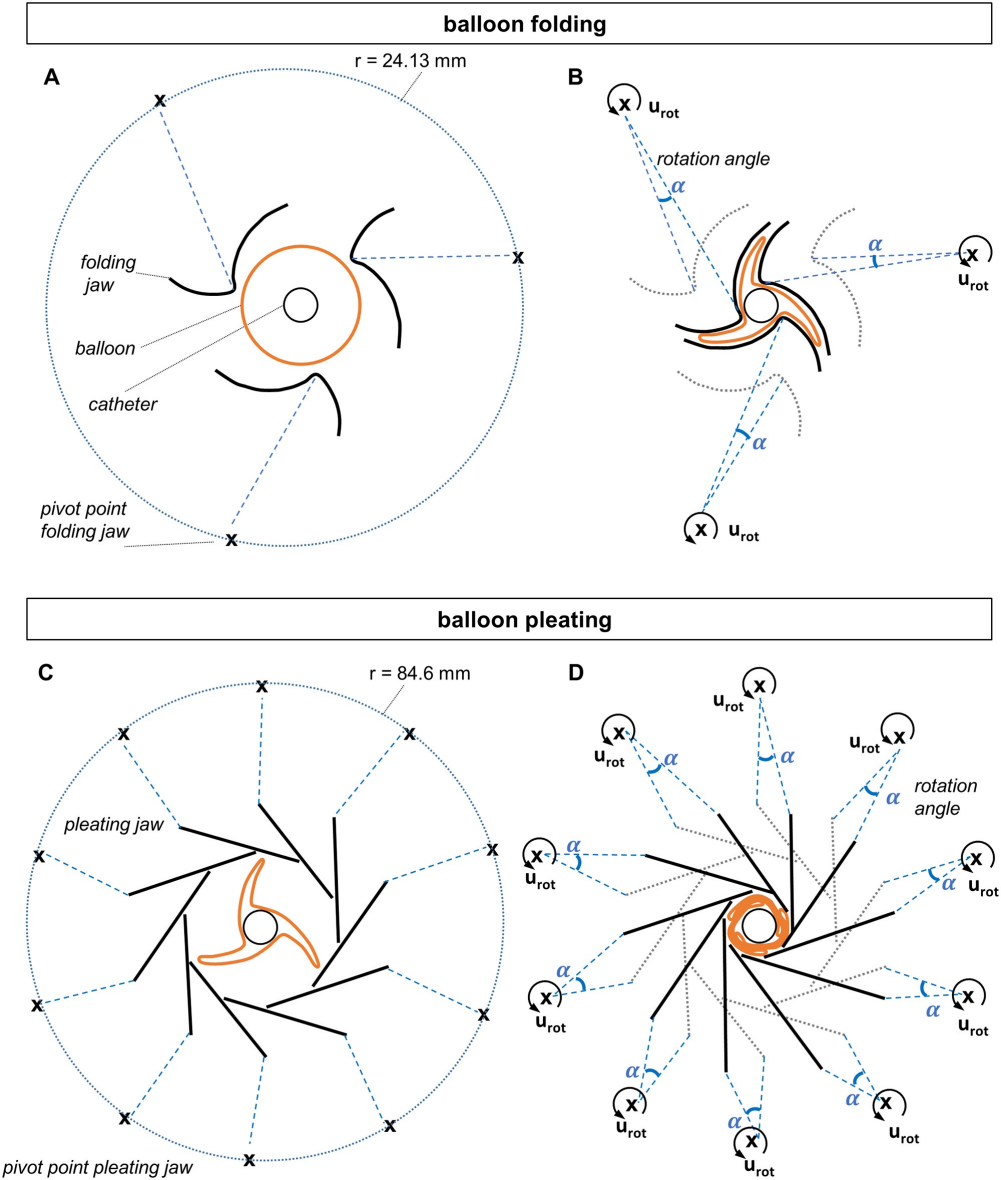
Schematic illustration of the boundary condition during the balloon folding simlation. A: Schematic illustration of the initial balloon and catheter inside the three folding jaws with the pivot point/RP of the jaws (indicated by “x”) which are connected to the jaws by a rigid body constraint (blue dashed line). B: Application of the rotation boundary condition *u_rot_* at the pivot point/RP of the folding jaws, which causes the jaws to rotate by the angle *α* and thus creates the three balloon folds. The initial position of the folding jaws is indicated with a dotted line. C: Schematic illustration of the folded balloon and catheter inside the ten pleating jaws with the pivot point/RP of the jaws (indicated by “x”) which are connected to the jaws by a rigid body constraint (blue dashed line). D: Application of the rotation boundary condition *u_rot_* at the pivot point/RP of the pleating jaws, which causes the jaws to rotate by the angle *α* and thus wraps the three balloon folds around the catheter. The initial position of the pleating jaws is drawn with a dotted line.

The boundary conditions of the balloon folding simulation are schematically illustrated in Fig 3 and S1 Fig. The folding and pleating jaws are connected by a rigid body constraint to their reference point (RP), which corresponds to the pivot point of the respective jaw. Rotation of the jaws is initiated by the rotation *u_rot_* of the respective RP. By rotating the three folding jaws by an angle *α*_fold_ of 4.58°, contact between the jaws and the balloon is established and thus a star-shaped balloon configuration is created within a step time *t* of 1 s (Fig 3B). Thereupon, vacuum is drawn by applying a slight negative pressure *p* of -0.1 MPa on the inner balloon surface to prevent the balloon from returning to its original shape. The folding jaws are then retracted by rotating the three folding jaws by an angle *α*_fold_ of -4.58° within a step time *t* of 1 s. In this way the contact between the jaws and the balloon is dissolved.

By rotating the ten pleating jaws by an angle *α*_pleat_ of -1.1° contact between the jaws and the balloon is established and thus the balloon folds are wrapped around the catheter (Fig 3D) within a step time *t* of 1 s. After retracting the pleating jaws by rotating the ten pleating jaws by an angle *α*_pleat_ of 1.1°, the contact between the jaws and the balloon is dissolved and the final folded balloon configuration achieved within a step time *t* of 1 s. During the entire folding simulation, the balloon is fixed at the proximal end, whereas displacement in axial direction is allowed to account for the balloon elongation. The catheter is fixed during the simulation to provide a kinematic limitation of the balloon folding.

During the entire folding process, contacts between balloon and folding/pleating jaws and self-contacts between the several balloon folds are considered by a friction coefficient *μ* of 0.2 (polyamid-steel).

The balloon is meshed using 170405 4-node quadrilateral M3D4 and 478 3-node triangle membrane elements M3D3 with an element size of about 0.037 mm (Fig 2). The folding/pleating jaws and the catheter are modeled as analytical rigids. The CPU time of the balloon folding simulation was approx. 8 CPU hours (Prozessor: Intel^®^ Xeon^®^ CPU E5-1660 v4 @ 3.20GHz 3.20 GHz).

#### Stent crimping and expansion simulations

To verify the broad applicability of the stent life-cycle simulation three different stent expansion scenarios were analysed: The detailed simulations of the life-cycle i) of a stent with homogenous stiffness (stent V1), ii) of a stent with heterogenous stiffness (stent V2) and iii) of an asymmetrically positioned stent V1 on the balloon catheter. Stent V1 represents a classical stent design with homogeneous strut widths *w_strut_* of 80 *μ*m. The strut widths of the last and penultimate rings of stent V2 were increased by 15% and 30% compared to V1, respectively, to provide higher radial strength at the stent ends aiming at a more homogeneous stent expansion. The remaining stent design parameters, e. g. strut thickness, position and type of connectors, geometry of the diamonds etc. correspond to stent design V1.

**Fig 4:**
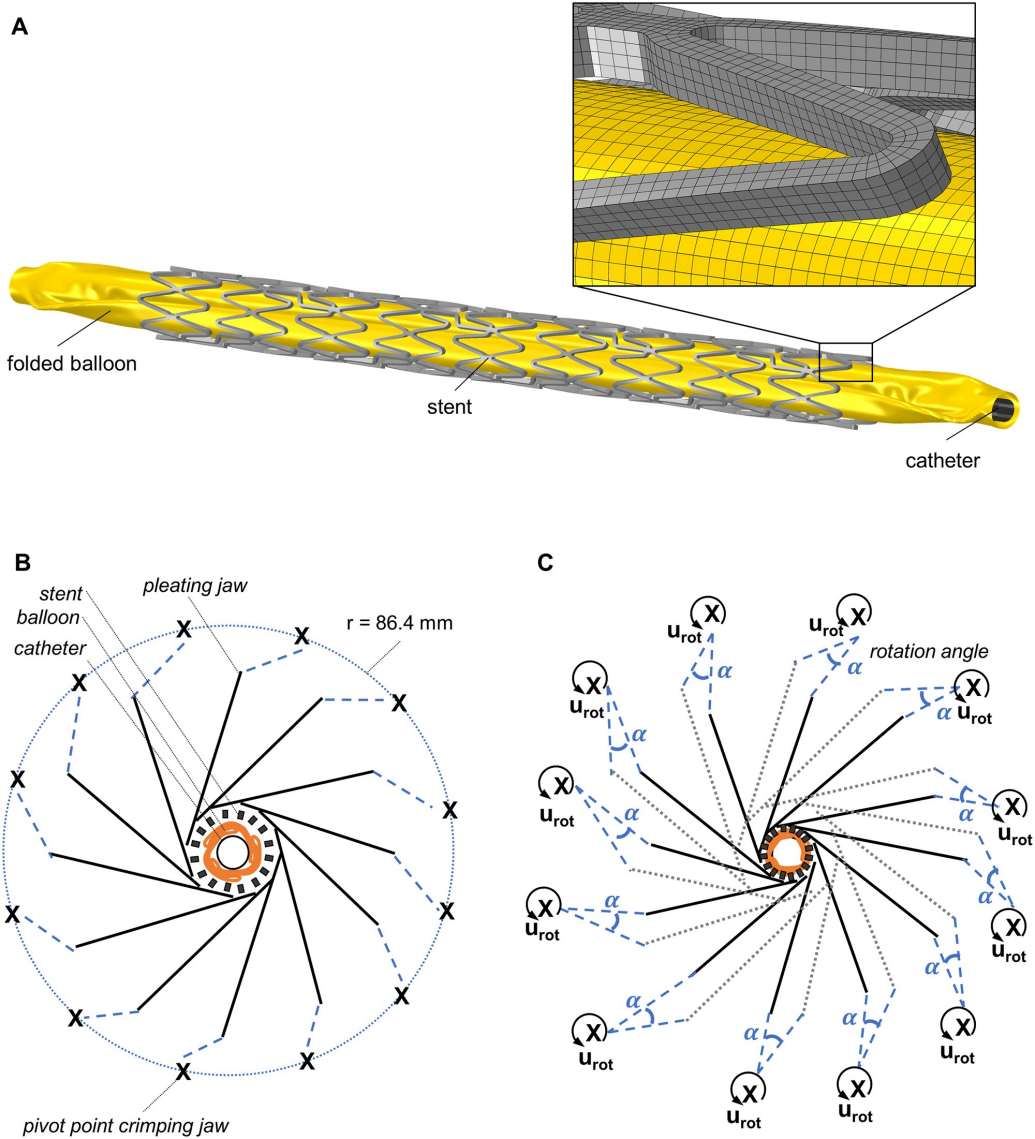
Model set-up of and schematic illustration of the boundary condition during the stent crimping simulation. A: Stent placed over the balloon-catheter system. B: Folded balloon, catheter and stent inside the twelve crimping jaws with the pivot point/RP of the jaws (indicated by “x”) and the connection of the jaws to the pivot points/RP via a rigid body constraint (blue dashed line). C: Application of the rotation boundary condition *u_rot_* at the pivot points/RP of the crimping jaws, which causes the jaws to rotate by the angle *α* and thus creates the three balloon folds.

After the stents were produced ready for use from the manufacturer (laser cut, stained, electropolished), they were first measured with a light microscope. In this way deviations due to post-processing steps, such as electropolishing, can be detected and subsequently considered in the simulation. Both stents have a length *L_stent_* of 19.54 mm, an initial outer diameter *D_stent_* of 1.97 mm and a strut thickness *t_strut_* of 0.12 mm. The folded balloon and catheter geometry are taken from the previous balloon folding simulation. The contact surfaces of the crimping jaws are modeled based on the measurements of the original in-house developed crimping machine. The pivot points of the crimping jaws are alligned on a circle with a radius *r* of 86.4 mm to the center of the crimping iris. The stent is centrally placed onto the balloon catheter system and inserted into the crimping iris consisting of twelve radially arranged crimping jaws (Fig 4). For the simulation of the asymmetrically positioned stent, the stent was moved 1 mm from the center to the distal end of the balloon.

The stent consists of surgical stainless steel 316L. The inelastic constitutive response of 316L is implemented through a von Mises plasticity material model with isotropic hardening (Eq 2) based on in-house experimental data from tensile tests on stent tubes. After reaching the tensile strength *R_m_*, strain hardening was considered by Hollomon’s power law equation (Eq 3). The material parameters used for modeling the constitutive material behavior of the stent (stainless steel 316L) are summarized in Table 2.

**Table 2:** Material parameters used for modeling the constitutive material behavior of the stent (stainless steel 316L).

Density *ρ*	Young’s modulus *E*	Yield strength R_p02_	Tensile strength R_m_	Poisson ratio *ν*	Strength coefficient *K*	Strain hardening parameter *n*
780 kg/m^3^	200 000 MPa	328 MPa	671 MPa	0.3	1440 MPa	0.39

The actual stent simulation consists of two phases: i) Stent crimping onto the balloon and ii) free stent-balloon expansion. For stent crimping, the crimping jaws are connected by a rigid body constraint to their RP corresponding to the respective pivot point. Rotation of the jaws is initiated by the rotation of their RP. The boundary conditions of the stent crimping simulation are schematically illustrated in Fig 4 and S2 Fig. By rotating the crimping jaws by an angle *α*_crimp_ of 0.464° contact bettween the stent and the jaws is initiated, the diameter of the crimping iris is reduced and the stent diameter compressed to 1.17 mm within a step time *t* of 3 s (Fig 4B and 4C). The jaws are then briefly held in position and a slight overpressure *p* of 0.1 MPa is applied to the inner balloon surface within a step time t of 0.0001 s. Thereupon, stent recoil is induced by retracting the crimping jaws and reducing the balloon pressure *p* to 0 MPa within a step time *t* of 1 s. The final stent expansion to an outer diameter of 3.74 mm is achieved by applying a linear increasing homogeneous pressure *p* up to 0.75 MPa on the inner side of the balloon within a step time *t* of 3.6 s. Finally, stent recoil is induced by decreasing the balloon pressure *p* to -0.1 MPa within a step time *t* of 0.4 s. During the entire simulation, the balloon is fixed at the proximal end, whereas a displacement in axial direction is possible at the distal end to allow for balloon elongation. The catheter is fixed during the simulation to provide a kinematic limitation of the stent crimping.

During the entire stent simulation, numerous contacts occur. In literature [8, 9], a global friction coefficient *μ* was considered neglecting the properties of several material pairings. Here, contacts between crimping jaws and stents as well as stent self-contact are considered with a friction coefficient *μ* of 0.1 (steel—steel). Stent-balloon, balloon-catheter and balloon self-contact are taken into account by a friction coefficient *μ* of 0.2 (polyamid-steel, polyamid-polyamid).

For stent discretization, eight node linear brick elements with reduced integration and hourglass control (ABAQUS element type C3D8R) were used. Due to the reudced integration points, these elements require lower computational effort. Compared to fully integrated elements reduced integrated elements are less/not susceptible to shear locking. Fully integrated elements show such susceptibility, making them too stiff in bending. To obtain accurate results despite the reduced number of integration points, four elements were defined over the stent thickness. To keep the analysis time reasonable, stent V1 is discretized by 153960 C3D8R elements and stent V2 by 133248 C3D8R elements with global element size of 0.03 mm. A sensitivity analyses was performed to ensure a sufficient mesh quality. The comparison of the simulation results with the adopted element size of 0.03 mm to a smaller element size of e.g. 0.015 mm (857996 elements) showed only minor deviations (e.g. a difference of about 1% with respect to radial displacement in the crimped state (S3 Fig)). However, using such a fine mesh size would increase the computational time by a factor of 2.5 (from approx. 44 CPU hours to 101 CPU hours, Prozessor: Intel^®^ Xeon^®^ CPU E5-1660 v4 @ 3.20GHz 3.20 GHz). With the adopted mesh size, CPU time for the crimping simulation was approx. 26 CPU-hours and for the stent expansion simulation approx. 18 CPU-hours.

### Stent expansion using a displacement-controlled simulation approach

The displacement-controlled (DC) approach is commonly used for stent expansion analysis and will be included in this study for comparison purpose. The starting point of the DC expansion is the crimped state of the stent V1 obtained from the detailed stent life-cycle simulation including all deformation and stress states. Thereby, the initial conditions of the DC expansion approach are identical with the stent life-cycle simulation. In the DC expansion approach, the presence of a folded balloon is neglected. Instead, a rigid expansion cylinder is positioned axially inside the stent. The cylinder is expanded to a final diameter *D* of 3.5 mm corresponding to the final balloon diameter D_bal_ in reality. After reaching the expansion diameter, the cylinder is contracted again to allow for stent recoil. During the expansion, contact between stent and expander is considered by a friction coefficient *μ* of 0.2 corresponding to the stent-balloon contact. The CPU time for the DC stent expansion simulation was approx. 2.75 CPU hours.

### Experimental procedure

The novelty of the present work consists in a comprehensive experimental validation of the free stent simulation. In the following, the experimental methods used to provide the data for validation are briefly described. This includes data concerning the folded balloon, the crimped/ expanded stent as well as the transient expansion behavior.

#### Angioplasty balloon

The semi-compliant Baroonda angioplasty balloon was received in the folded configuration from the manufacturer. The balloon was measured under the light microscope both in the folded and unfolded state. For the folding pattern validation, a *μ*CT (Phönix v|tome|xs 240/180 research edition, GE Sensing & Inspection Technologies, Wunstorf, Germany) image of a folded balloon was taken. In addition, the folded balloon was embedded in epoxy resin, subsequently sliced and imaged under the light microscope to illustrate the folded balloon cross-section.

#### Stent

The two different stent designs V1 and V2 were laser cut from 2 mm diameter tubes with a wall thickness *t_wall_* of 0.12 mm from 316L surgical stainless steel. The material properties of the stents were derived from uniaxial tensile test data of the initial tube provided by the manufacturer. To determine the initial stent geometry, the stents were measured under a light microscope. To analyze the crimped stent dimensions, a total of eight stents were reproducibly crimped using an in-house developed crimping machine based on the iris principle. The final crimped stent dimensions were then measured under the light microscope. For the basic validation of the crimping process, a *μ*CT scan of stent V1 and sections of an embedded stent V1 were exemplary prepared analogous to the folded balloon. Thereupon, the crimped stents were reproducibly expanded by a in-house developed pump unit. The pump unit reproducibly replicates the pressurization via hand pump as it is performed by the surgeon to allow for a most realistic stent expansion behavior. Stent expansion is recorded with a high-speed camera (HCC-1000, VDS^®^ Vosskühler, Osnabrück, Deutschland). The images were automatically evaluated using a in-house Matlab script to determine the DB ratio. Therefore, the stent was first segmented from the background using a threshold function and thereupon the stent diameter was determined on the basis of the number of pixels. Care was taken to ensure that the high-speed camera was aligned perpendicular to the stent expansion plane in order to ensure that the DB ratio was determined as accurately as possible and to reduce measurement errors.

To determine the final stent dimension in the expanded stent, eight expanded stents were measured under the light microscope. Furthermore, scanning electron microscopy (SEM) (1450 VP, Leo Elektronenmikroskopie GmbH, Oberkochen, Germany) images of an expanded V1 stent were taken for the validation of deformation and stress states due to the onset of flow and plastification processes in the stent material.

## Results and discussion

In the following, the results from the validation of the numerical simulations are presented and subsequently discussed. The experimental data are listed in more detail in the supporting information (S1–S4 Tables).

### Balloon folding simulation

**Fig 5:**
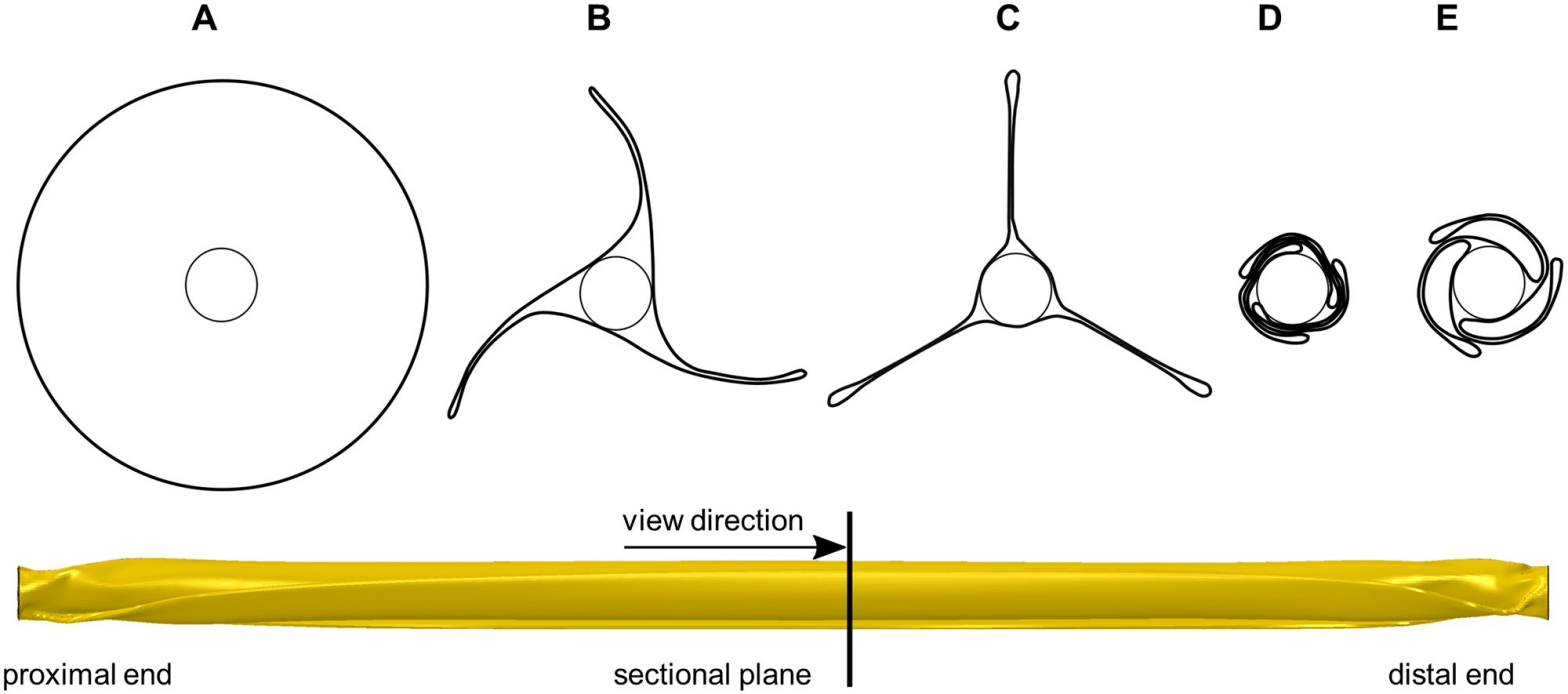
Successive simulation results of the balloon folding process. A: Balloon cross section of the initial cylindrical balloon. B: Cross section after the folding process. C: Cross section after the vacuum generation and retraction of the folding jaws. D: Cross section after the pleating process. E: Cross section of the final folded balloon configuration after retraction of the pleating jaws.

Fig 5 shows the successful folding process consisting of the generation of the three folds and their subsequent wrapping around the catheter. The predicted folded balloon has an overall length *L_bal,fold_* of 27.44 mm and a diameter *D_bal,fold_* of 1.5 mm.

**Fig 6:**
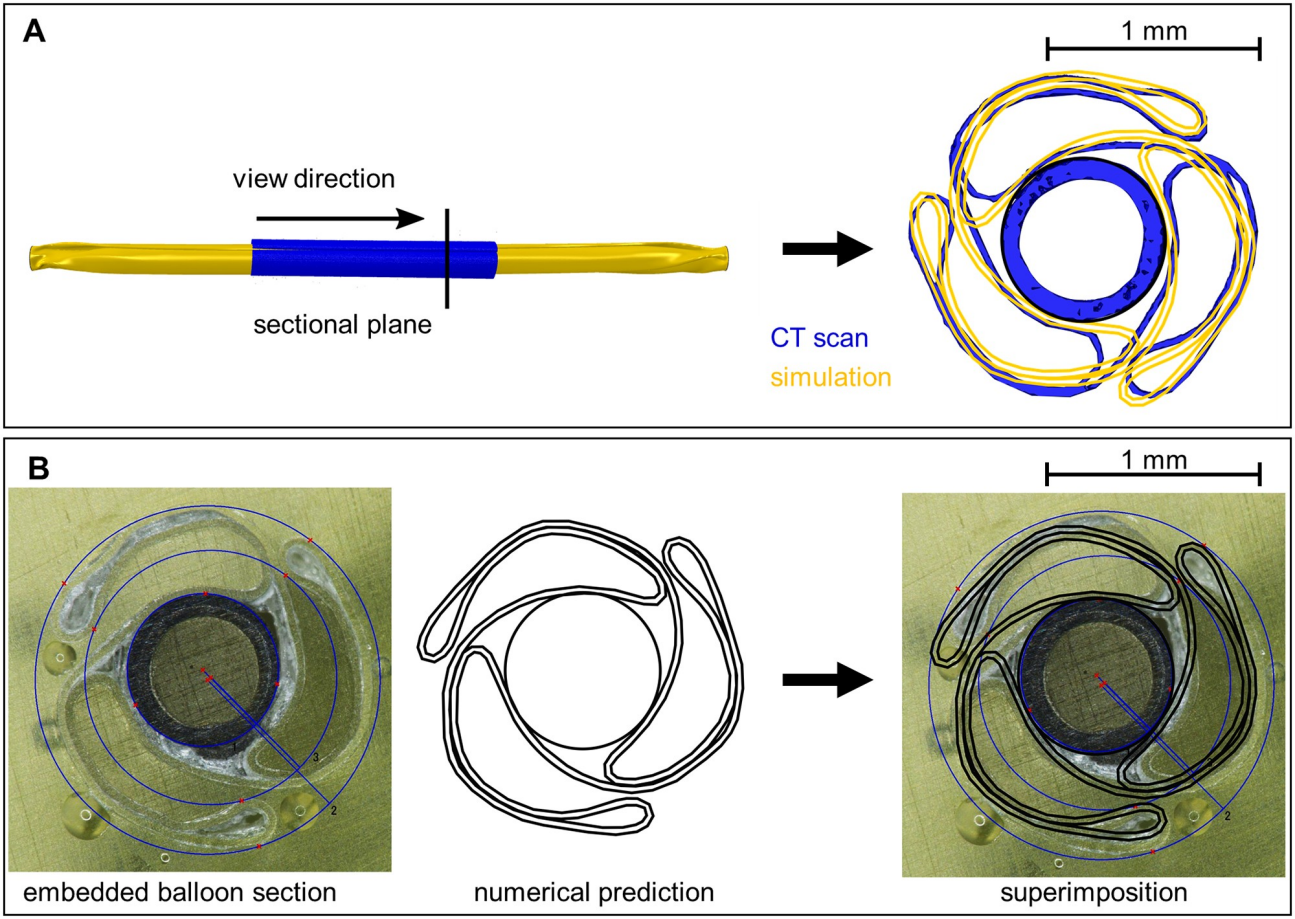
Validation of the balloon folding simulation. A: Superimposition of a section of the predicted folded balloon (yellow) with CT Data (blue). B: Superimposition of a section of the predicted folded balloon (black) with a section of an embedded balloon.

The superposition of the simulation results with a CT scan (Fig 6A) and an embedded section of a balloon (Fig 6B) shows that the basic shape of the balloon folding can be predicted in the simulation. The superimposition of the predicted and embedded balloon sections display only a minimal deviation (Fig 6B). In comparison to the CT scan, the predicted balloon shows a slightly increased compression in the center (Fig 6A). This is possibly due to a slight relaxation of the balloon material over time in reality. However, in the subsequent crimping process, the balloon diameter is reduced again compensating for the slightly more compressed balloon folds in the simulation. As a result, the balloon diameter deviation is not expected to have a significant effect on the subsequent simulation results.

### Crimping simulation

**Fig 7:**
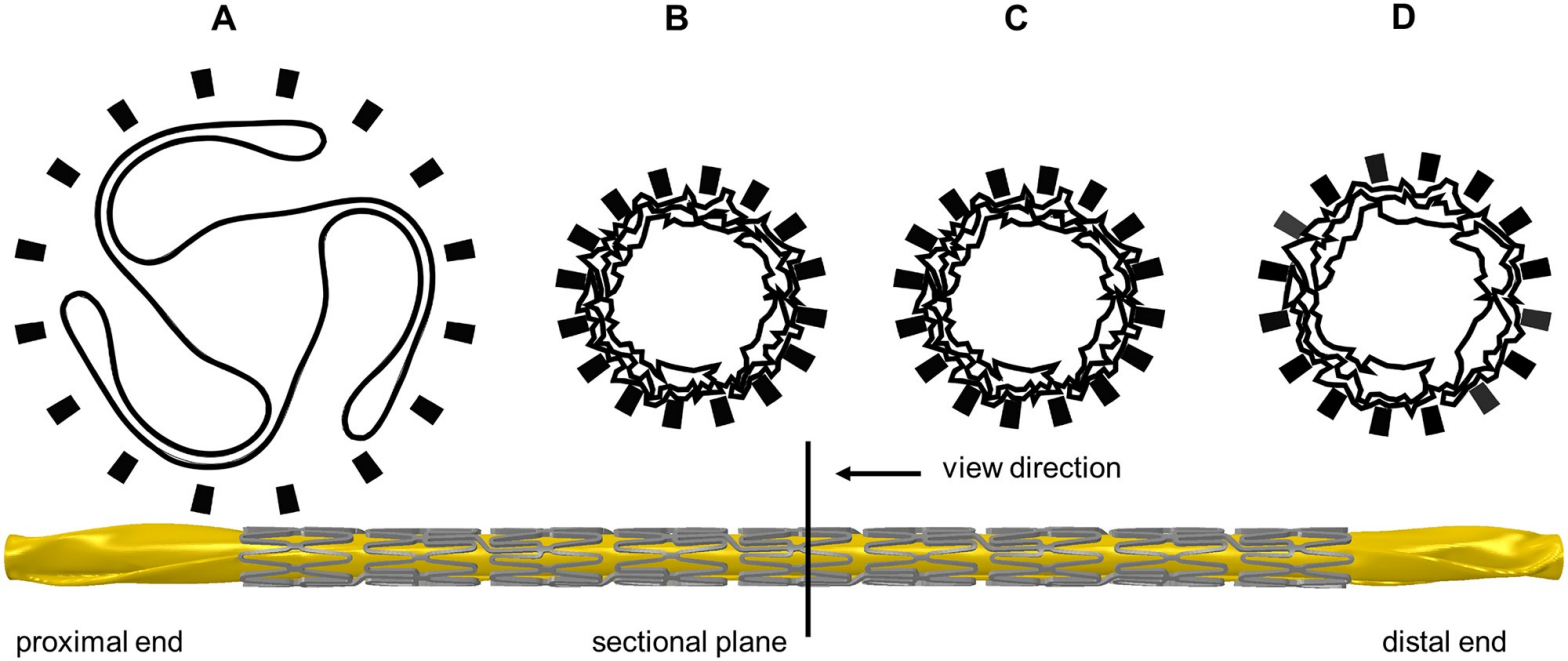
Successive simulation results of the stent crimiping process. A: Initial balloon-stent configuration before crimping. B: Maximum compressed stent configuration after rotating the crimp jaws (not visualized) around their pivot point and thereby reducing the diameter of the crimp iris. C: Holding phase. D: Final crimped stent-balloon configuration after retraction of the crimping jaws and stent recoil.

**Fig 8:**
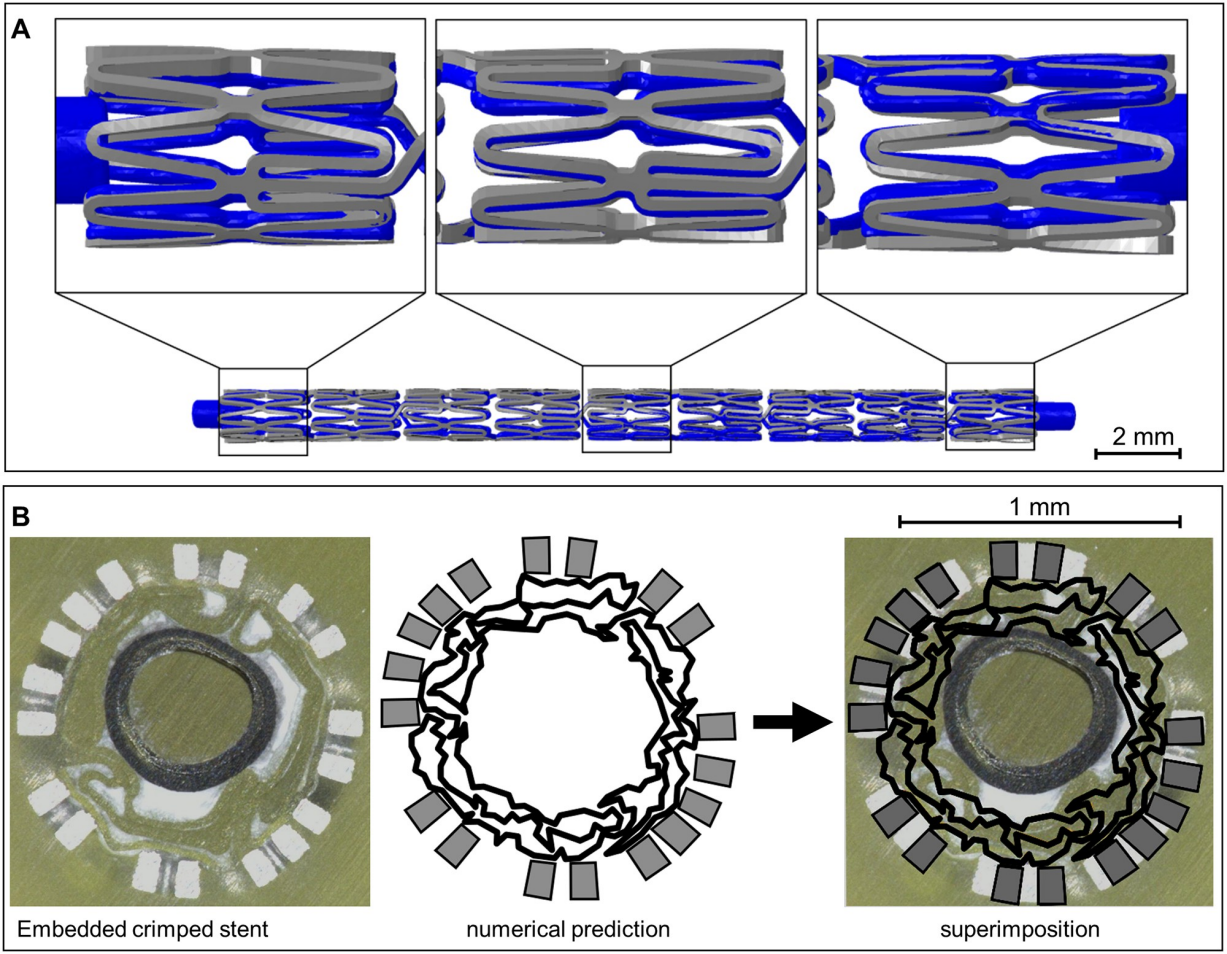
Validation of the crimping simulation. A: Comparison of the numerical results of stent crimping (grey) with CT data of a crimped stent (blue). B: Superimposition of the numerical results of stent crimping (black) with the section of an embedded stent.

Fig 7 shows the successive crimping process of the stent-balloon system. The final stent diameter *D_crimp_* and length *L_crimp_* are very consistent with experimental measurements with a maximum deviation < 1%, but still within the range of the standard deviation (Table 3). The superimposition of the predicted crimped stent with an CT scan shows very good agreement with nearly congruent stent struts and connectors (Fig 8A). The small deviations in the superposition are potentially due to minor deviations in the initial stent geometry (simulation: statistical representative stent geometry, CT Scan: exemplary stent geometry) or a slight bending of the stent during the fixation in the CT. The wrinkled shape of the balloon folds after crimping is accurately captured as evident from the superimposition of the simulated with an embedded crimped stent (Fig 8B). As far as the authors know, this is the first simulation to depict this phenomenon. In 2006, Wang et al. [19] experimentally proved the wrinkled balloon shape after crimping but neglected this phenomenon in their simulation. Thus, it can be assumed that the simulation presented here is the most realistic approach for mapping stent crimping so far.

**Table 3:** Validation of the predicted stent dimensions after crimping with experimental measurements.

	Stent V1			Stent V2		
	Sim. [mm]	Exp. [mm]	SD	Sim. [mm]	Exp. [mm]	SD
D_crimp_	1.20	1.19	± 0.02	1.197	1.19	± 0.01
L_crimp_	19.70	19.75	± 0.05	19.69	19.78	± 0.16

### Free stent expansion simulation

**Fig 9:**
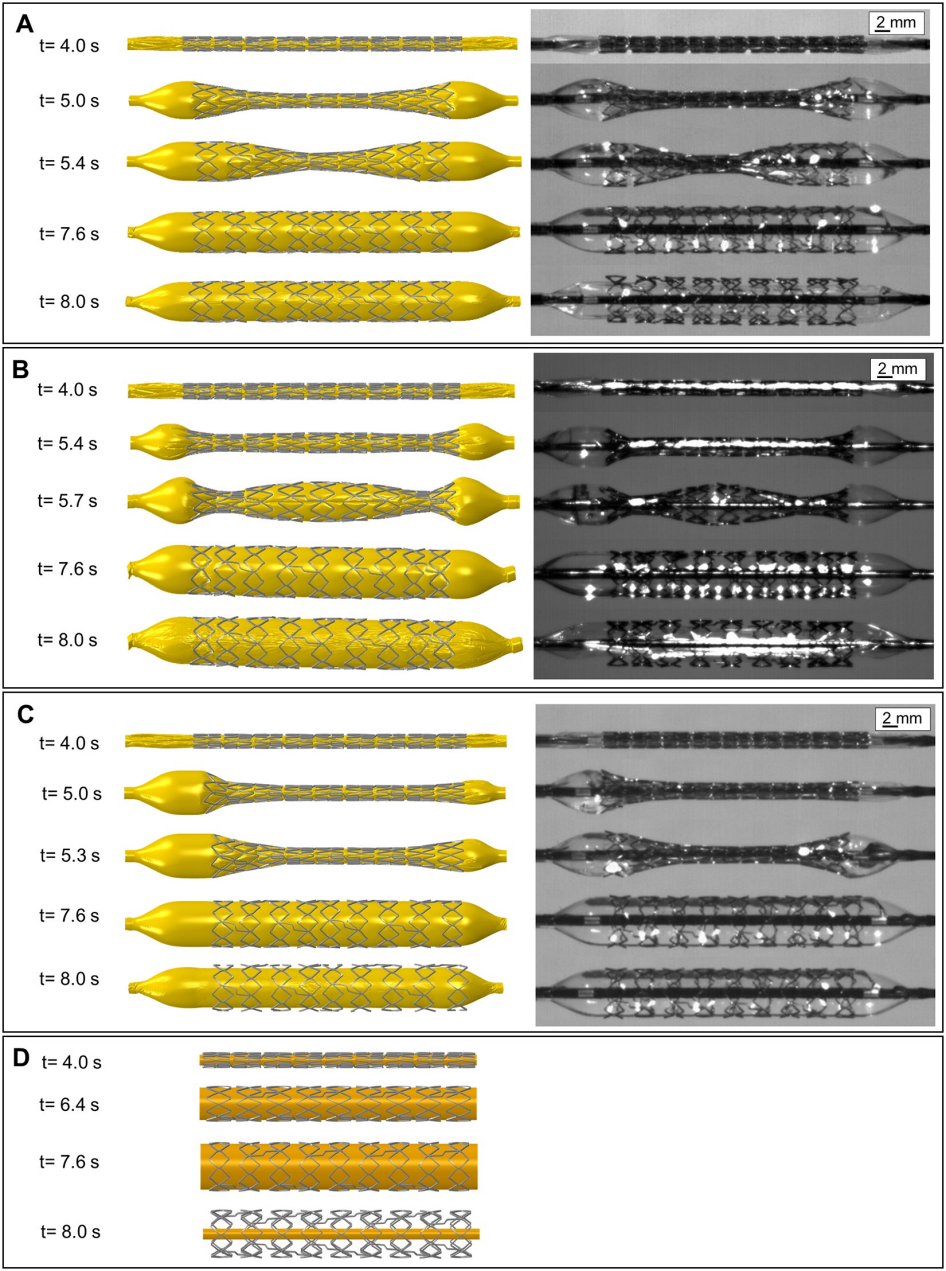
Comparison of the predicted stent expansion behavior (left) with high-speed recordings (right). A: Detailed stent life-cycle simulation of stent V1. B: Detailed stent life-cycle simulation of stent V2. C: Detailed stent life-cycle simulation of stent V1 with an assymmetric positioning of the stent on the balloon catheter D: Displacement-controlled expansion of stent V1. For reasons of conformity with Fig 10, the given time labels were related to the entire simulation of the stent life-cycle. Therefore, stent expansion starts at a step time *t* of 4 s.

The predicted transient stent expansion from the detailed stent life-cycle simulation shows good agreement with high-speed camera images. The transient stent expansion behavior of stent V1 corresponds to the typical stent expansion pattern with pronounced DB ratio (Fig 9A). Stent V2, shows a rather untypical spindle-shaped stent expansion behavior before reaching the final cylindrical expanded configuration (Fig 9B). The influence of the asymmetric positioning of the stent on the balloon catheter was also successfully predicted in the simulation. Due to the asymmetric positioning, the DB ratio is initially pronounced at the distal end. This can be attributed to the larger free balloon length at the distal end and thus to a greater wedge effect of the balloon when pressurized. Slight deviations in the predicted expansion behavior are potentially due to the positioning of the stent, since the exact positioning of the stent in the experimental analysis is not known. The DC expansion simulation of stent V1, however, shows a continuous cylindrical expansion. In this way, the final expanded stent shape can be predicted, but no information about the stent expansion behavior can be obtained (Fig 9C).

**Fig 10:**
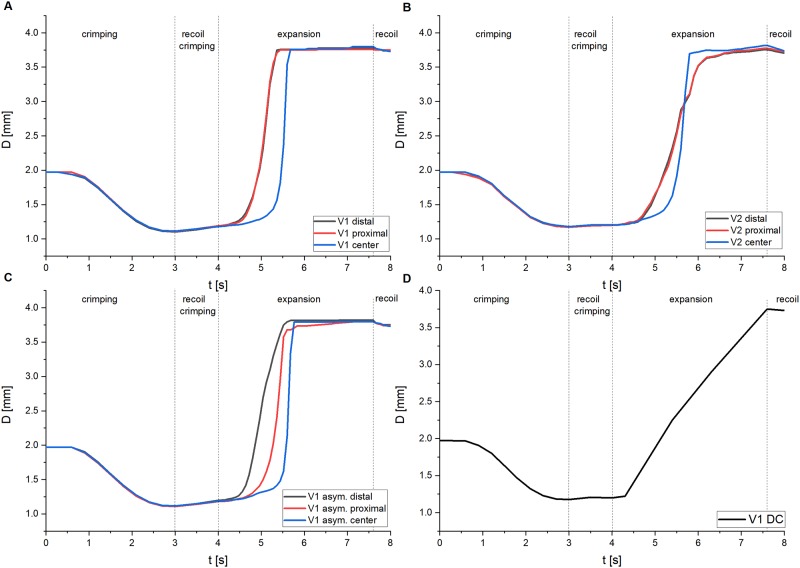
Numerically determined diameter course within the simulation of the entire stent life-cycle. A: Detailed stent life-cycle simulation of stent V1. B: Detailed stent life-cycle simulation of stent V2. C: Detailed stent life-cycle simulation of an asymmetrical positioned stent V1. D: Displacement-controlled expansion of stent V1. Within the evaluation of the results from the detailed simulation approach (A, B, C), a distinction was also made between the diameter progression of the stent ends (red) and the central part of the stent.

Stent expansion behavior was further evaluated in terms of the development of the stent diameter *D* during the entire stent life-cycle simulation (Fig 10). During crimping (*t* = 0–3 s) and recoil after crimping (*t* = 3–4 s), the diameters of stent V1 and V2 are very similar. During stent expansion (*t* = 4–8 s), the diameter of stent V1 initially increases at the ends and reaches the final state after about 1 s (*t* = 5 s). Diameter change in the central area is delayed by about 1 s, whereby the final stent diameter is reached here after an expansion time of 1.5 s (*t* = 5.5 s) (Fig 10A). Similar to stent V1, the diameter of stent V2 initially increases at the ends, but with a smaller gradient due to the increased stiffness. The increase in diameter in the central area of stent V2 is also delayed by about 1 s, but is subsequently accelerated. After an expansion time of about 1.5 s (*t* = 5.5 s) the same diameter is achieved at the ends and in the central area. However, the final stent diameter in stent V2 is reached in the central area about 0.5 s before the stent ends (Fig 10B). The diameter curve of the asymmetrically positioned stent V1 is very similar to that of the stent V1. Due to the asymmetric positioning, however, there are different free balloon lengths, which cause a time delay in the diameter increase at the distal and proximal ends. A larger free balloon length is present at the distal end. As a result, the increase in diameter is initially more pronounced at the distal end, whereas the increase in diameter at the proximal end is delayed by approx. 0.5 s (Fig 10C). In contrast to the detailed simulation approach, the DC approach results in a constant, linear increase of the diameter over the entire stent (Fig 10D).

The predicted dimensions of stents V1, V2 and the DC expanded stent V1 at maximum expansion and after recoil show good agreement with experimental measurements (Table 4). The highest discrepancy is obtained for the stent length *L* with a maximum error < 2%. This deviation is potentially due to different balloon adhesion in simulation and reality. In reality, the stent is pressed very strongly into the balloon during crimping. Thereby, the balloon is locally compressed increasing adhesion. This effect can only be partially numerically considered by the static friction coefficient *μ*. Due to this increased contact, the stent has a higher longitudinal resistance against the wedge effect of the balloon ends during the initial stent expansion and might be therefore, less compressed in the longitudinal direction. However, since the deviation is limited to a value of 1–2%, the results can still be classified as good. According to [8], a more realistic modeling would be possible by using a dynamic coefficient of friction which considers e.g. the rate of relative movement or the surface finish.

**Table 4:** Validation of the predicted stent dimensions after stent expansion with experimental measurements (mean value and standard deviation(SD)).

**Stent V1—detailed stent life-cycle simulation**			
	Sim.	Exp. (mean)	SD
D_exp_	3.80 mm	3.78	± 0.03 mm
D_recoil,exp_	3.73 mm	3.71	± 0.05 mm
L_exp_	18.0 mm	18.35	± 0.13 mm
L_recoil,exp_	18.20 mm	18.54	± 0.13 mm
DB_prox_	0.62	0.63	± 0.02 mm
DB_dist_	0.62	0.62	± 0.02 mm
**Stent V2—detailed stent life-cycle simulation**			
	Sim.	Exp. (mean)	SD
D_exp_	3.77 mm	3.75	± 0.07 mm
D_recoil,exp_	3.71 mm	3.70	± 0.07 mm
L_exp_	18.76 mm	19.10	± 0.14 mm
L_recoil,exp_	18.65 mm	19.02	± 0.16 mm
DB_prox_	-0.20	-0.21	± 0.05
DB_dist_	-0.18	-0.21	± 0.03
**Stent V1—Displacement-controlled expansion approach**			
	Sim.	Exp. (mean)	SD
D_exp_	3.77 mm	3.78	± 0.03 mm
D_recoil,exp_	3.67 mm	3.71	± 0.05 mm
L_exp_	18.77 mm	18.35	± 0.13 mm
L_recoil,exp_	18.78 mm	18.54	± 0.13 mm
DB_prox_	0	0.63	± 0.02 mm
DB_dist_	0	0.62	± 0.02 mm

In comparison to the detailed stent life-cycle simulation, DC expansion approach leads to slightly larger deviations with respect to deformed stent shape. The DC approach represents an ideal stent expansion. Thereby, changes in stent length are only due to the radial displacement and stretching of the stent diamonds. In reality, however, due to the presence of the balloon, the stent is first compressed longitudinally by the DB effect before being stretched again at maximum balloon expansion. These successive compression and elongation effects cannot be considered within the DC approach which might lead to greater deviation in stent length at maximum stent expansion.

**Fig 11:**
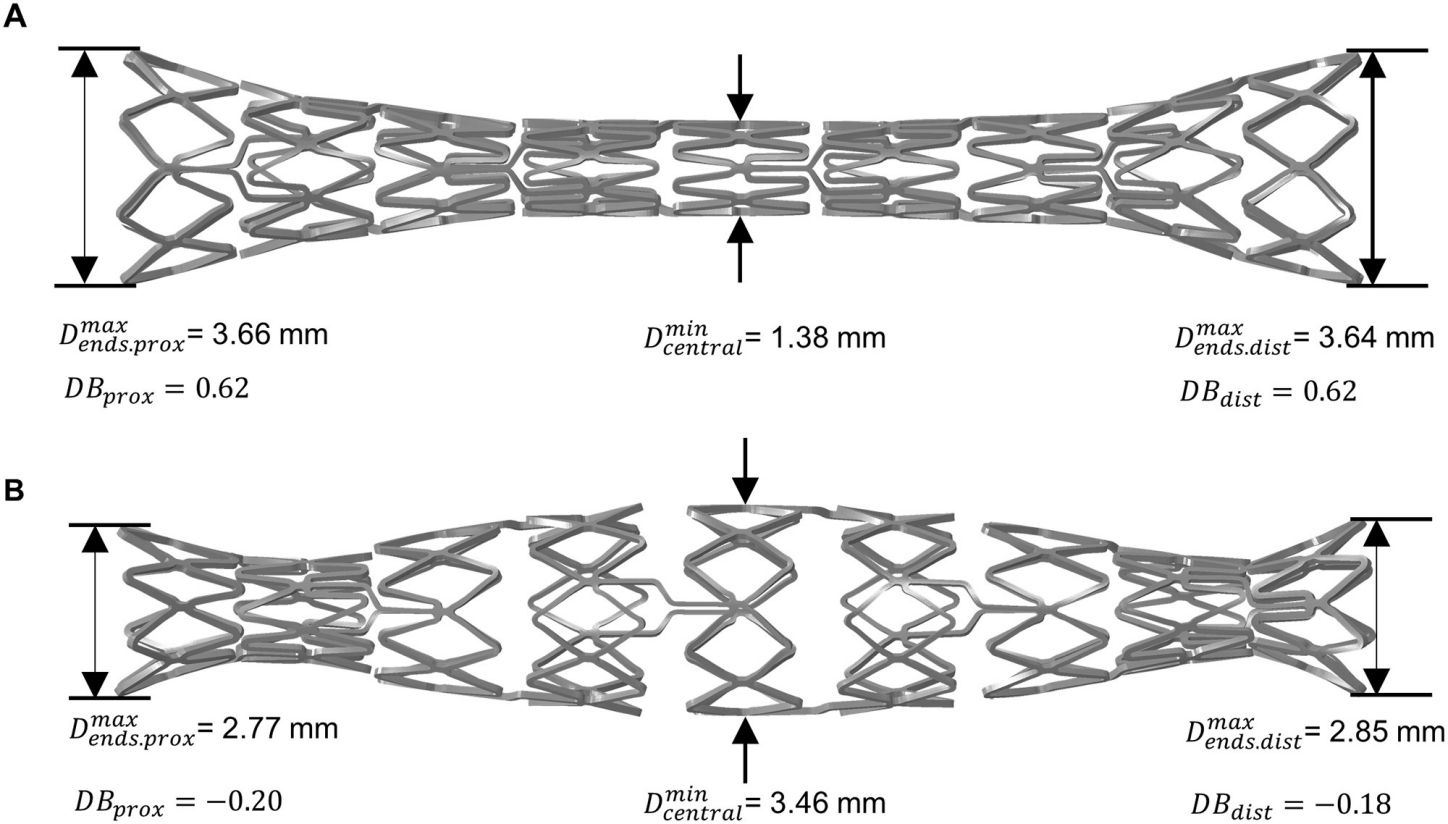
Determination of the dogboning ratio for stent within the stent life-cycle simulation. A: Stent V1 with a typical pronounced dogboning ratio, B: Stent V2 with a rather untypical dogboning ratio due to the heterogenous stent design.

DB ratio was determined using the maximum stent diameter at the proximal *D_end,prox_* and distal *D_end,dist_* ends as well as the minimum central stent diameter *D_central_* (Fig 11, Eq 1) and subsequently compared with experimental data (S3 Table). The predicted DB ratio of stent V1 is within the standard deviation of the experimentally determined DB ratios during free stent expansion (Table 4, Fig 11A). In contrast to stent V1, stent V2 shows a rather uncharacteristic spindle-like expansion behavior. Nevertheless, DB ratio was evaluated based on Eq 1 to validate the transient expansion behavior of stent V2. This results in a negative DB ratio of -0.21 due to increased expansion in the central part of the stent compared to its ends. Again, the predicted DB ratio of stent V2 is within standard deviation of the experimental data (Table 4, Fig 11B). As far as the authors know, this is the first quantitative approach to successfully validate the extent of the DB effect determined in the simulation. Since DC expansion does not replicate the transient expansion behavior, DB ratio could not be determined.

**Fig 12:**
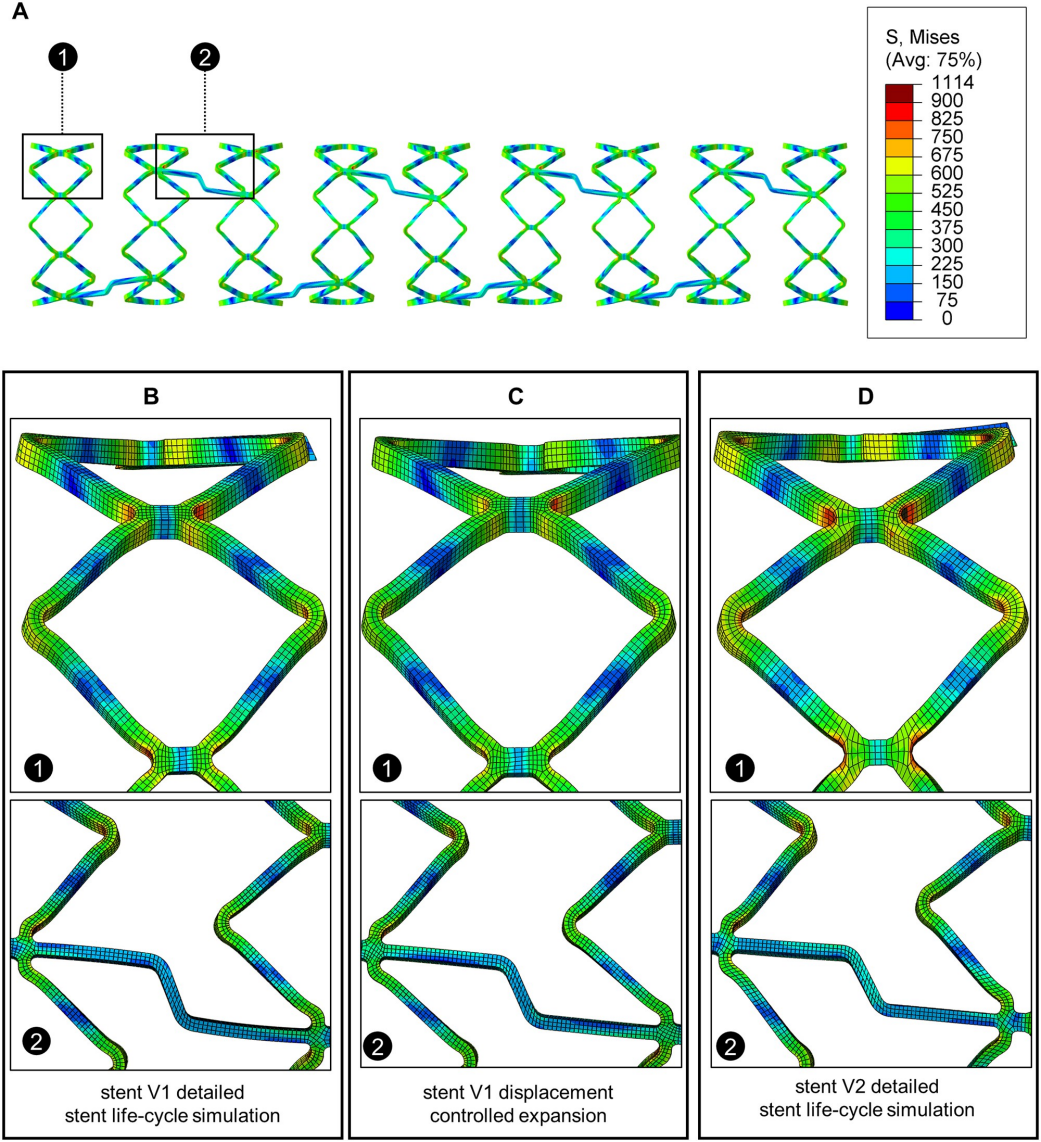
Comparison of stress distribution in the maximum expanded state. A: Overview of the stress distribution and localization of the following close-up representations. B: Stress distribution obtained from the detailed stent life-cycle simulation of stent V1. C: Stress distribution obtained from the displacement controlled expansion simulation of stent V1. D: Stress distribution obtained from the detailed stent life-cycle simulation of stent V2.

Finally, the stress distribution obtained from the DC expansion approach was compared with the stent life-cycle simulation of stent V1 and V2 in the maximum expanded state (Fig 12). Approximately equal stress distribution and peaks are obtained at the stent diamonds for both simulation approaches for stent V1 (Fig 12B① and 12C①). In the area of the connectors (Fig 12B② and 12C②), slightly higher stresses tend to occur in the DC approach. Despite these small deviations, the DC approach and the detailed stent life-cycle simulation of stent V1 result in approximately the same stress distribution in the maximum expanded stent state. Due to the lower complexity and computation time the DC approach is recommended for the analysis of the maximum occurring stresses. Stent V2 shows basically a similar stress distribution, but with slightly higher stresses in the area of the stent diamonds (Fig 12D①).

**Fig 13:**
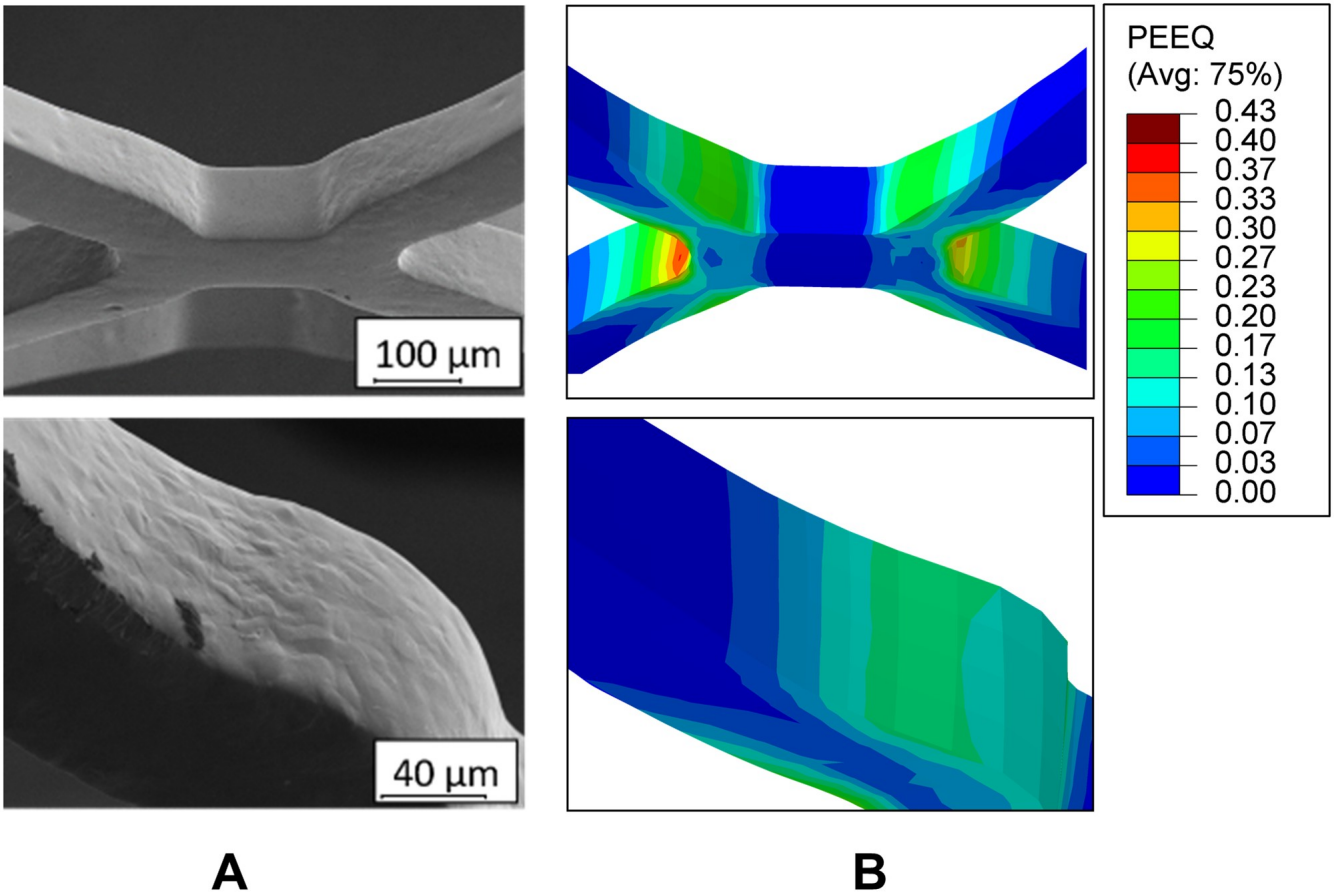
Qualitative validation of stent deformation with scanning electron microscope images of an expanded V1 stent. A: Scanning electron microscope images and B: Plastic equivalent strain distribution resulting from the detailed simulation of stent V1.

Deformation analysis shows that the highest values occur at the bending elements during stent expansion. The deformation distribution obtained from the detailed stent life-cycle simulation of stent V1 is qualitatively compared to SEM of an expanded stent. The comparison of the deformation values with SEM does not allow for a quantitative validation of the deformation. However, within SEM, local flow processes and thus plastification of the material can be identified, which in turn can be qualitatively compared with the occurring deformation or stresses. In the areas of low stress/deformations (*σ* < *σ*_*yield*_ = 328 MPa) a smooth surface is observed due to electro polishing and the absence of plastic deformation. In the areas of higher stress/deformations (*σ* > *σ*_*yield*_ = 328 MPa), the material begins to flow and deforms plastically resulting in wavy areas as apparent in the corresponding SEM images (Fig 13).

### Limitations and remarks

The simulation methodology presented in this paper allows to represent the stent life-cycle numerically, which was proven by the quantitative and qualitative validation of the numerical results with experimental data. Nevertheless, some limitations remain to which the (partly relatively small) deviations from from experimental observations can be attributed. The limitations relate to stent geometry, material and contact modeling, balloon deployment strategy, as well as the absence of the analysis of interaction with the blood vessel wall.

In contrast to previous analyses, the stent dimensions were not idealized but are based on averaged measurement results of the respective stent. Thus, the influence of rework steps, such as pickling and electro polishing, can be considered. However, a direct stent reconstruction based on CT data was not considered. Therefore, the stent geometries used in these simulations are to be regarded as statistically representative geometries.

A von Mises plasticity model with isotropic hardening based on a quasi-static uniaxial tensile test on a stent tube was used to model the mechanical behavior of the stent. The influence of the strain rate $\dot{∊}$ is not included in this model, which may underestimate the yield strength *R_p02_*. However, experimental studies in literature [29] have shown that the strain rate $\dot{∊}$ has only a very small influence on the mechanical behavior of 316L. During stent expansion, strain rates $\dot{∊}$ of up to 0.5 s^-1^ occur. According to [29], yield strength *R_p02_* and tensile strength *R_m_* at this strain rate $\dot{∊}$ are approximately identical to the quasi-static case. The assumption of quasi-static material behavior is therefore justified. However, slight deviations in the material behavior cannot be guaranteed. A further limitation of the material modeling can be attributed to the loading history of the stent. During crimping and expansion, the stent is first subjected to compression during crimping and then to tension. A plastic pre-deformation on tension or compression results in a lower yield *R_p02_* for metallic materials with a plastic deformation in the opposite direction compared to the pre-deformation. This phenomenon is known as the Bauschinger effect. The Bauschinger effect is not considered within the material model during stent simulation. Due to the reduced yield strength, the Bauschinger effect might potentially lead to a reduction of the required expansion pressure. An effect on transient expansion behavior is, however, unlikely.

Furthermore, stent expansion is achieved by a linear increase of uniform pressure on inner surface of the balloon whereas in reality, stent expansion results from injecting a fluid into the balloon catheter. For a more realistic simulation, a complex fluid-structure interaction (FSI) problem has to be solved during which the mesh of the balloon lumen has to adapt very strongly during balloon deployment. Such a detailed simulation approach was not aimed at in the context of this work. However, the experimental validation of the free stent expansion simulation results based on the linear pressurization of the balloon interior already showed very realistic results.

The results presented here have shown that even minor stent design modifications influence the stent expansion behavior. The spindle-shaped expansion of stent V2 can be attributed to the increased radial stiffness due to the increased strut widths at the stent ends. Since the free expansion was analysed, only minor conclusions can be drawn about possible stent-artery interactions. It is assumed that the reduction of the DB ratio resulting in a more homogeneous stent expansion is potentially beneficial regarding the reduction of high wall stress/strains during the implantation. However, the modeling of the stent-artery interaction is essential to verify this assumption and to exactly determine the extent of possible vascular injuries. Furthermore, coronary arteries are located on the outside of the heart, causing the stent to bend slightly during the implantation. To represent the stent expansion as realistically as possible, the numerical model should further be extended by the presence of a curved vessel.

Finally, the effects of stent design and balloon catheter (heterogeneous or homogeneous design) on expansion behavior are briefly discussed. The main cause of the DB ratio, and hence the transient boundary curvature, is due to the unrestricted balloon expansion at the ends. This assumption is based, among other things, on the analysis of an asymmetric positioned stent. Due to the varying free balloon length, a different DB ratio occured at the proximal and distal stent end. During stent deployment, balloon expansion is unrestricted at the ends, whereas in the central area it is constrained by the stent. As balloon pressure increases, expansion can eventually continue toward the center, resulting in the stent DB ratio. To completely prevent this effect, a stent with optimum longitudinal stiffness would be required which better absorbs the radial variations due to the uneven inflation pressure of the balloon. However, the provision of such a stent was not the focus of this work. Rather, the simulation of a stent design with atypical expansion behavior should prove the broad applicability of the simulation approach. In further studies, an expansion-optimized stent could be developed on the basis of the presented stimulation method. In addition to the optimization of the stent design, the expansion behavior of the stent might also be improved by modifying the balloon design/material. By increasing the stiffness of the outer balloon portion, the expansion there could be limited and thus the strong initial expansion of the balloon ends could be reduced. In addition, a change in the free balloon length, e.g. a shortening, could affect the extent of the DB ratio. However, the free balloon length can only be reduced to a certain extent, otherwise the stent might be dislocated from the balloon catheter during expansion. The investigation of the influence of the free balloon length on the stent could be the subject of further studies.

## Conclusions

A numerical simulation approach for the detailed stent life-cycle simulation has been successfully developed, including balloon folding, stent crimping and the expansion of the stent-balloon-system expansion. Experimental validation of the individual simulation steps showed consistently very good agreement. In particular, the transient free stent expansion could be reproduced realistically, especially with regard to the DB effect development. The successful simulation of two stent design, one with pronounced and one with reduced DB ratio, as well as an asymmetrically positioned stent further demonstrated the universal applicability of the simulation approach for variable stent geometries and positioning on the balloon catheter. The comparison of the simulation results with the commonly used simplified DC expansion approach has shown that the DC approach cannot realistically represent the transient stent expansion, but provides similar results regarding the stress distribution in the maximum expanded stent state. Therefore, it can be concluded that the detailed stent life-cycle simulation is essential for the analysis/optimization of the stent expansion behavior The use of the DC stent expansion approach is in return justified for a fast and resource-saving stress analysis. Despite the limitation on material modeling or lack of interaction of the stent with the arterial wall, a promising numerical approach has been presented which can be used for studies and improvements of stent designs.

## Supporting information

S1 Fig Overview of the individual simulation steps for the simulation of balloon folding. Application and release of rotatory boundary conditions, pressure boundary conditions, contact conditions in the individual simulation steps.
(TIF)

S2 Fig Overview of the individual simulation steps for the simulation of stent crimping and expansion simulation. Application and release of rotatory boundary conditions, pressure boundary conditions, contact conditions in the individual simulation steps.
(TIF)

S3 Fig Visual representation of the adopted mesh quality. Comparison of A: von Mises stress stress distribution at the maximum expanded state and B: radial displacement at the crimped state for a global stent mesh size of 0.03 mm and 0.015 mm.
(TIF)

S1 Table Overview of the experimental determined dimensions of stent design V1. (PDF)

S2 Table Overview of the experimental determined dogboning ratio of stent V1. (PDF)

S3 Table Overview of the experimental determined dimensions of stent design V2. (PDF)

S4 Table Overview of the experimental determined dogboning ratio of stent V2. (PDF)

## References

[1] World Health Organization. Global Health Estimates 2016: Deaths by Cause, Age, Sex by Country and by Region 2000-2016; 2018. Available from: http://www.who.int/news-room/fact-sheets/detail/the-top-10-causes-of-death.

[2] Farb A, Sangiorgi G, Carter AJ, Walley VM, Edwards WD, Schwartz RS, et al Pathology of Acute and Chronic Coronary Stenting in Humans. Circulation. 1999;99(1):44–52. 10.1161/01.cir.99.1.44 9884378

[3] Kim MS, Dean LS. In-Stent Restenosis. Cardiovascular Therapeutics. 2011;29(3):190–198. 10.1111/j.1755-5922.2010.00155.x 20406239

[4] Kaiser C, Brunner-La Rocca HP, Buser PT, Bonetti PO, Osswald S, Linka A, et al Incremental cost-effectiveness of drug-eluting stents compared with a third-generation bare-metal stent in a real-world setting: randomised Basel Stent Kosten Effektivitäts Trial (BASKET). The Lancet. 2005;366(9489):921–929. 10.1016/S0140-6736(05)67221-2 16154019

[5] Moses JW, Leon MB, Popma JJ, Fitzgerald PJ, Holmes DR, O’Shaughnessy C, et al Sirolimus-eluting stents versus standard stents in patients with stenosis in a native coronary artery. The New England journal of medicine. 2003;349(14):1315–1323. 10.1056/NEJMoa035071 14523139

[6] Morice MC, Serruys PW, Sousa JE, Fajadet J, Ban Hayashi E, Perin M, et al A randomized comparison of a sirolimus-eluting stent with a standard stent for coronary revascularization. The New England journal of medicine. 2002;346(23):1773–1780. 10.1056/NEJMoa012843 12050336

[7] Lepor NE, Madyoon H, Kereiakes DJ. Effective and efficient strategies for coronary revascularization in the drug-eluting stent era. Reviews in cardiovascular medicine. 2002;3 Suppl 5:S38–50. 12478234

[8] De Beule M, Mortier P, Carlier SG, Verhegghe B, van Impe R, Verdonck P. Realistic finite element-based stent design: The impact of balloon folding. Journal of Biomechanics. 2008;41(2):383–389. 10.1016/j.jbiomech.2007.08.014 17920068

[9] Bukala J, Kwiatkowski P, Malachowski J. Numerical analysis of crimping and inflation process of balloon-expandable coronary stent using implicit solution. International journal for numerical methods in biomedical engineering. 2017;33(12). 10.1002/cnm.2890 28425201

[10] Dumoulin C, Cochelin B. Mechanical behaviour modelling of balloon-expandable stents. Journal of Biomechanics. 2000;33(11):1461–1470. 10.1016/s0021-9290(00)00098-1 10940405

[11] Lally C, Dolan F, Prendergast PJ. Cardiovascular stent design and vessel stresses: A finite element analysis. Journal of Biomechanics. 2005;38(8):1574–1581. 10.1016/j.jbiomech.2004.07.022 15958213

[12] Migliavacca F, Petrini L, Colombo M, Auricchio F, Pietrabissa R. Mechanical behavior of coronary stents investigated through the finite element method. Journal of Biomechanics. 2002;35(6):803–811. 10.1016/s0021-9290(02)00033-7 12021000

[13] Auricchio F, Constantinescu A, Conti M, Scalet G. A computational approach for the lifetime prediction of cardiovascular balloon-expandable stents. International Journal of Fatigue. 2015;75:69–79. 10.1016/j.ijfatigue.2015.02.002

[14] Wang Q, Fang G, Zhao Y, Wang G, Cai T. Computational and experimental investigation into mechanical performances of Poly-L-Lactide Acid (PLLA) coronary stents. Journal of the mechanical behavior of biomedical materials. 2017;65:415–427. 10.1016/j.jmbbm.2016.08.033 27643678

[15] Zhao S, Gu L, Froemming SR. On the importance of modeling stent procedure for predicting arterial mechanics. Journal of Biomechanical Engineering. 2012;134(12):121005 1–6. 10.1115/1.4023094 23363207

[16] David Chua SN, MacDonald BJ, Hashmi MSJ. Effects of varying slotted tube (stent) geometry on its expansion behaviour using finite element method. Journal of Materials Processing Technology. 2004;155-156:1764–1771. 10.1016/j.jmatprotec.2004.04.395

[17] Debusschere N, Segers P, Dubruel P, Verhegghe B, de Beule M. A finite element strategy to investigate the free expansion behaviour of a biodegradable polymeric stent. Journal of Biomechanics. 2015;48(10):2012–2018. 10.1016/j.jbiomech.2015.03.024 25907549

[18] Kiousis DE, Wulff AR, Holzapfel GA. Experimental studies and numerical analysis of the inflation and interaction of vascular balloon catheter-stent systems. Annals of Biomedical Engineering. 2009;37(2):315–330. 10.1007/s10439-008-9606-9 19048377

[19] Wang WQ, Liang DK, Yang DZ, Qi M. Analysis of the transient expansion behavior and design optimization of coronary stents by finite element method. Journal of Biomechanics. 2006;39(1):21–32. 10.1016/j.jbiomech.2004.11.003 16271584

[20] Zahedmanesh H, John Kelly D, Lally C. Simulation of a balloon expandable stent in a realistic coronary artery-Determination of the optimum modelling strategy. Journal of Biomechanics. 2010;43(11):2126–2132. 10.1016/j.jbiomech.2010.03.050 20452594

[21] Schiavone A, Qiu TY, Zhao LG. Crimping and deployment of metallic and polymeric stents—finite element modelling. Vessel Plus. 2017;1(1). 10.20517/2574-1209.2016.03

[22] Migliavacca F, Petrini L, Montanari V, Quagliana I, Auricchio F, Dubini G. A predictive study of the mechanical behaviour of coronary stents by computer modelling. Medical engineering & physics. 2005;27(1):13–18. 15604000 10.1016/j.medengphy.2004.08.012

[23] Etave F, Finet G, Boivin M, Boyer JC, Rioufol G, Thollet G. Mechanical properties of coronary stents determined by using finite element analysis. Journal of Biomechanics. 2001;34(8):1065–1075. 10.1016/s0021-9290(01)00026-4 11448698

[24] Auricchio F, Di Loretto M, SACCO E. Finite-element Analysis of a Stenotic Artery Revascularization Through a Stent Insertion. Computer methods in biomechanics and biomedical engineering. 2001;4(3):249–263. 10.1080/10255840108908007

[25] Gervaso F, Capelli C, Petrini L, Lattanzio S, Di Virgilio L, Migliavacca F. On the effects of different strategies in modelling balloon-expandable stenting by means of finite element method. Journal of Biomechanics. 2008;41(6):1206–1212. 10.1016/j.jbiomech.2008.01.027 18374340

[26] Kim DB, Choi H, Joo SM, Kim HK, Shin JH, Hwang MH, et al A comparative reliability and performance study of different stent designs in terms of mechanical properties: foreshortening, recoil, radial force, and flexibility. Artificial organs. 2013;37(4):368–379. 10.1111/aor.12001 23461583

[27] Chua SND, Mac Donald BJ, Hashmi MSJ. Finite-element simulation of stent expansion. Journal of Materials Processing Technology. 2002;120(1-3):335–340. 10.1016/S0924-0136(01)01127-X

[28] SIMULIA Abaqus. Abaqus Theory Manual 6.13. Simulia; 2013.

[29] Langdon GS, Schleyer GK. Unusual strain rate sensitive behaviour of AISI 316L austenitic stainless steel. The Journal of Strain Analysis for Engineering Design. 2004;39(1):71–86. 10.1177/030932470403900106

