# Supplementary figures and images for: Experimentally validated simulation of coronary stents considering different dogboning ratios and asymmetric stent positioning
Source: PLoS One. 2019 Oct 18;14(10):e0224026. doi: 10.1371/journal.pone.0224026 (PMC6799901; doi:10.1371/journal.pone.0224026)

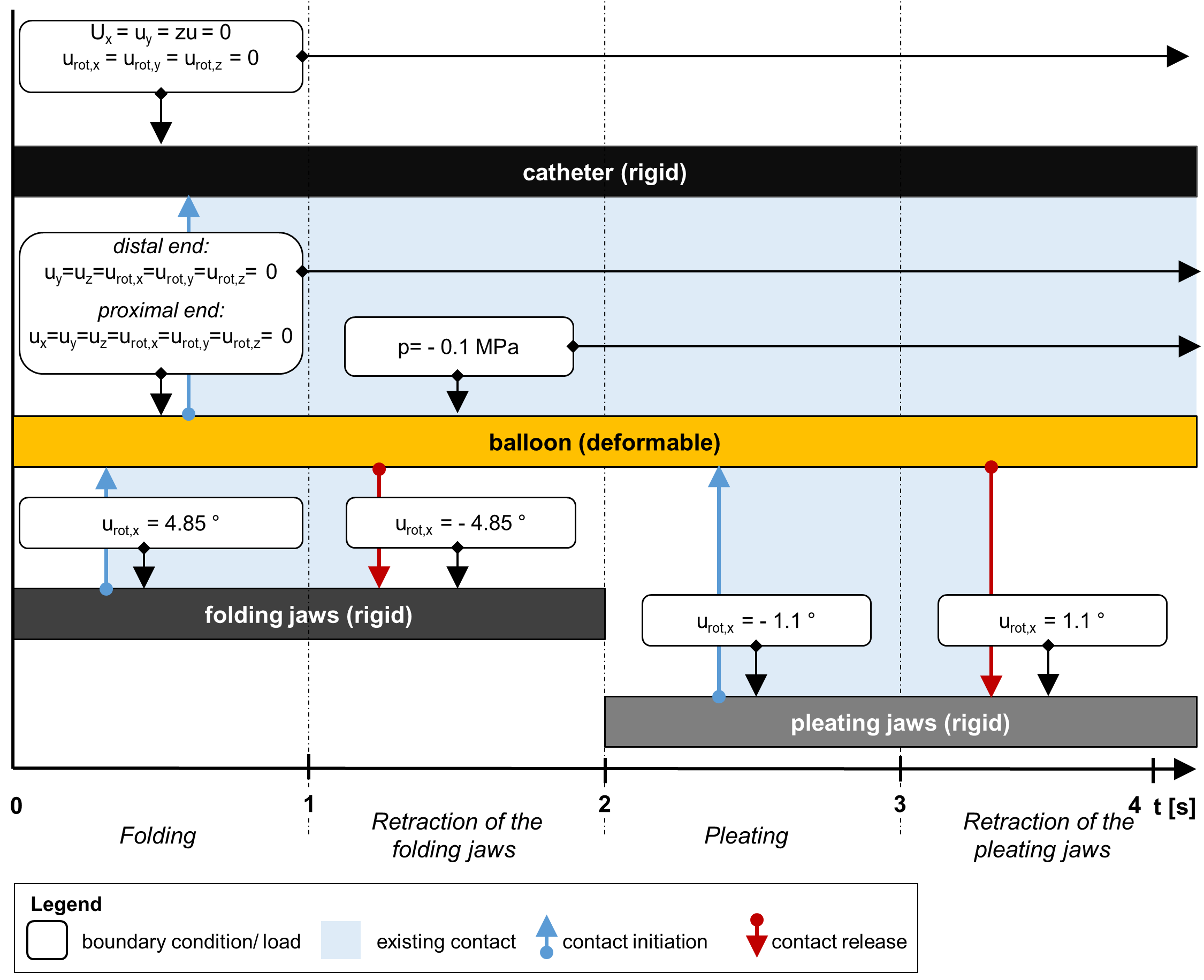

Supplement: S1 Fig — Application and release of rotatory boundary conditions, pressure boundary conditions, contact conditions in the individual simulation steps. (TIF) [file pone.0224026.s001.tif]

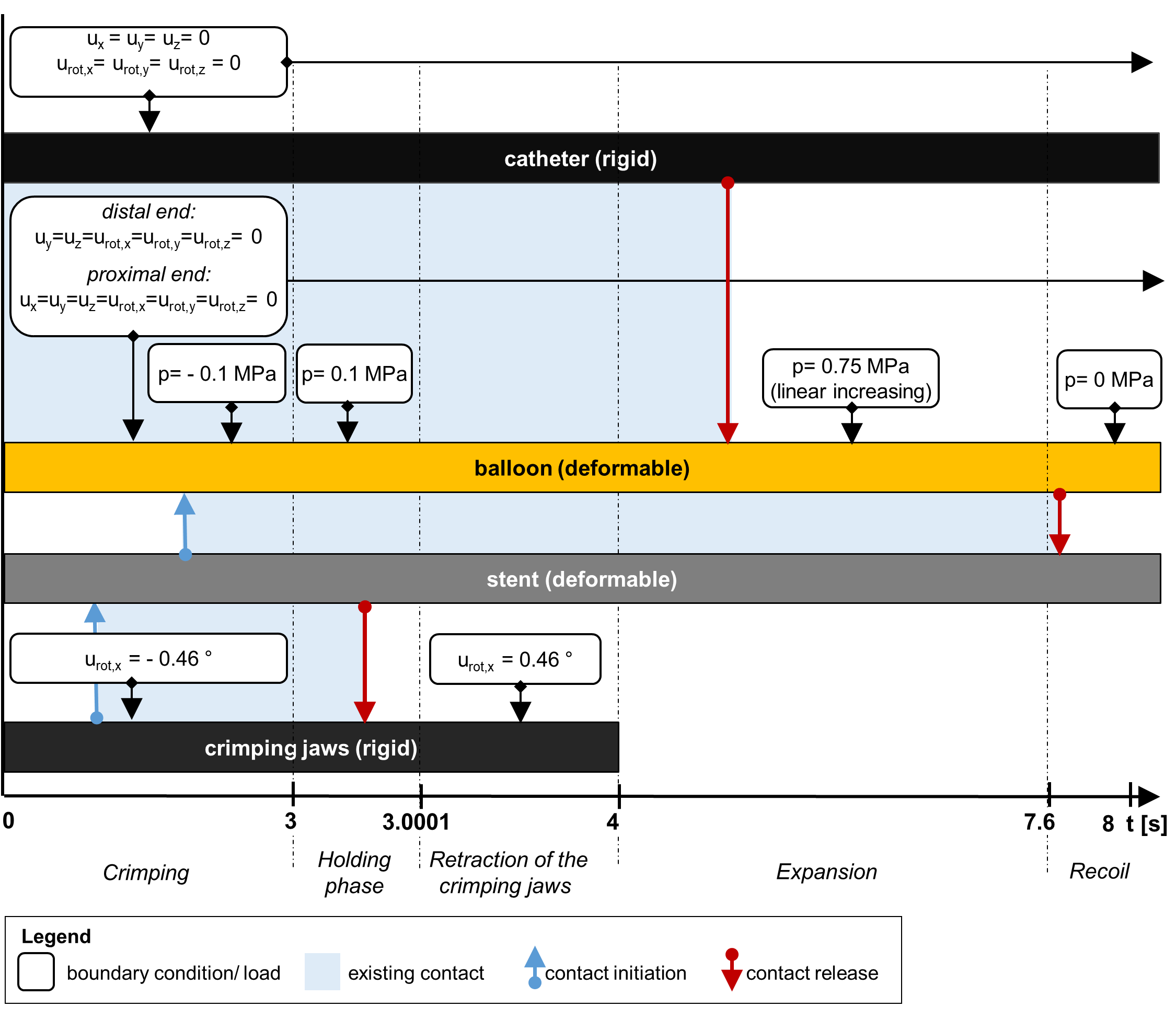

Supplement: S2 Fig — Application and release of rotatory boundary conditions, pressure boundary conditions, contact conditions in the individual simulation steps. (TIF) [file pone.0224026.s002.tif]

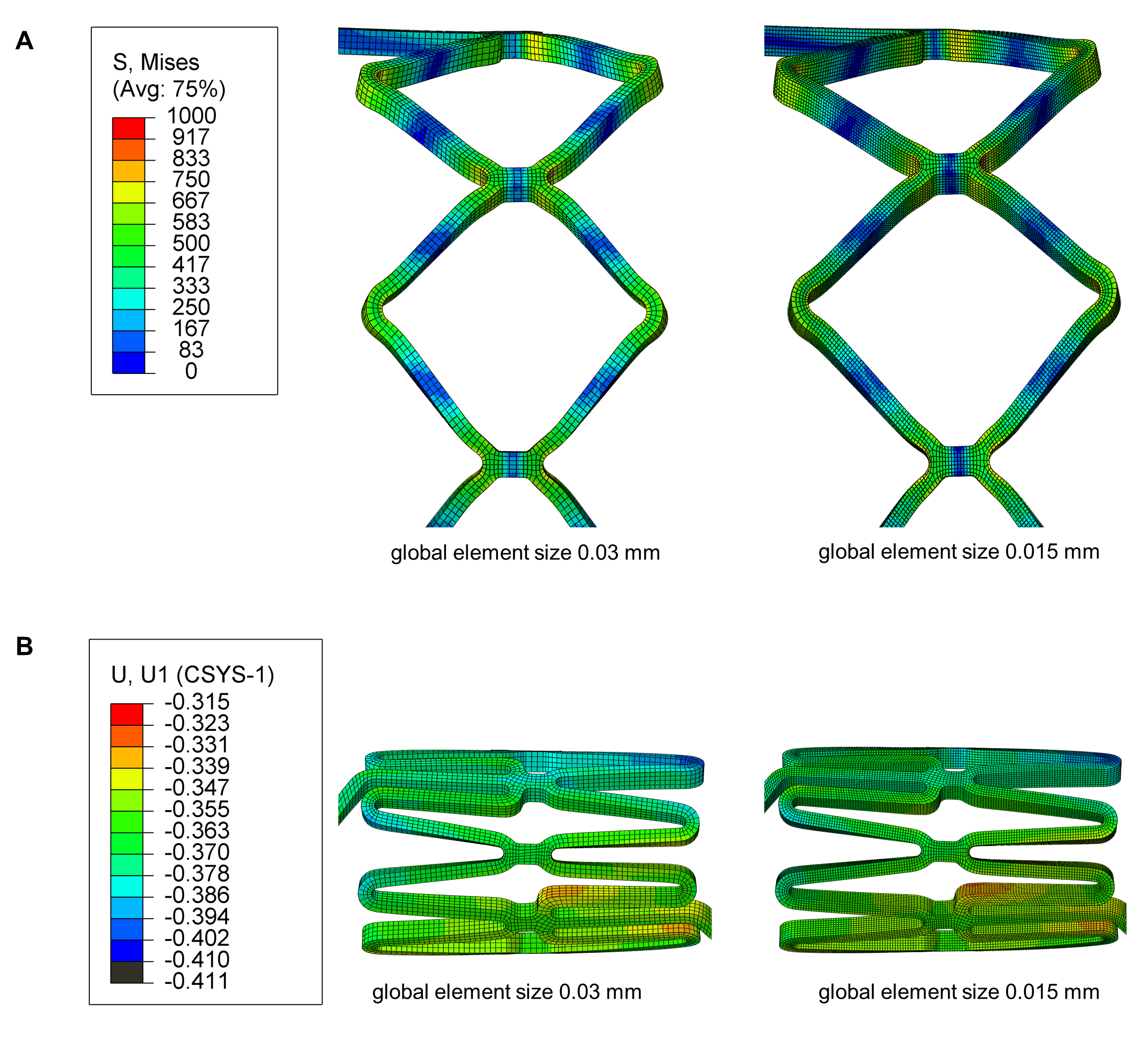

Supplement: S3 Fig — Comparison of A: von Mises stress stress distribution at the maximum expanded state and B: radial displacement at the crimped state for a global stent mesh size of 0.03 mm and 0.015 mm. (TIF) [file pone.0224026.s003.tif]
