# Supplementary material for: Experimentally validated simulation of coronary stents considering different dogboning ratios and asymmetric stent positioning
Source: PLoS One. 2019 Oct 18;14(10):e0224026. doi: 10.1371/journal.pone.0224026 (PMC6799901; doi:10.1371/journal.pone.0224026)
Supplement: S1 Table — (PDF) [file pone.0224026.s004.pdf]

Stent sample	Stent V1 length L [mm]			Stent V1 diameter D [mm]		
	L_{crimp}	L_{exp}	$L_{recoil,exp}$	D_{crimp}	D_{exp}	$D_{recoil,exp}$
Stent V1 01	19.77	18.55	18.84	1.17	3.74	3.74
Stent V1 02	19.82	18.41	18.41	1.18	3.78	3.64
Stent V1 03	19.79	18.41	18.62	1.18	3.80	3.72
Stent V1 04	19.70	18.21	18.42	1.21	3.77	3.77
Stent V1 05	19.65	18.12	18.48	1.19	3.80	3.65
Stent V1 06	19.73	18.32	18.55	1.20	3.74	3.74
Stent V1 07	19.80	18.32	18.47	1.22	3.82	3.74
Stent V1 08	19.75	18.48	18.55	1.21	3.80	3.65
Mean	19.75	18.35	18.54	1.19	3.78	3.71
SD ±	0.05	0.13	0.13	0.02	0.03	0.05

L_{crimp}: stent length after crimping
L_{exp}: stent length at maximum balloon expansion
$L_{recoil,exp}$: stent length after recoil
D_{crimp}: stent diameter after crimping
D_{exp}: stent diameter at maximum balloon expansion
$D_{recoil,exp}$: stent diameter after recoil
