# Supplementary material for: Experimentally validated simulation of coronary stents considering different dogboning ratios and asymmetric stent positioning
Source: PLoS One. 2019 Oct 18;14(10):e0224026. doi: 10.1371/journal.pone.0224026 (PMC6799901; doi:10.1371/journal.pone.0224026)
Supplement: S2 Table — (PDF) [file pone.0224026.s005.pdf]

Stent sample	Dogboning DB [—]		Stent V1 diameter D_{DB} at Dogboning [mm]		
	DB_{prox}	DB_{dist}	$D_{prox,DB}$	$D_{mid,DB}$	$D_{dist,DB}$
Stent V1 01	0.65	0.62	3.45	1.22	3.24
Stent V1 02	0.60	0.60	3.21	1.28	3.21
Stent V1 03	0.65	0.66	3.44	1.22	3.58
Stent V1 04	0.60	0.60	3.41	1.35	3.41
Stent V1 05	0.61	0.61	3.15	1.22	3.15
Stent V1 06	0.63	0.63	3.28	1.22	3.28
Stent V1 07	0.66	0.65	3.36	1.14	3.28
Stent V1 08	0.63	0.58	3.44	1.29	3.08
Mean	0.63	0.62	3.34	1.24	3.28
SD ±	0.02	0.02	0.11	0.06	0.15

Analysis of the dogboning ratio DB based on Eq. 1

DB_{dist}: dogboning ratio at distal stent end

DB_{prox}: dogboning ratio at proximal stent

D_{DB}: corresponding stent diameter for the analysis of the dogboning ratio
