# Supplementary material for: Experimentally validated simulation of coronary stents considering different dogboning ratios and asymmetric stent positioning
Source: PLoS One. 2019 Oct 18;14(10):e0224026. doi: 10.1371/journal.pone.0224026 (PMC6799901; doi:10.1371/journal.pone.0224026)
Supplement: S3 Table — (PDF) [file pone.0224026.s006.pdf]

Stent sample	Stent V2 length L [mm]			Stent V2 diameter D [mm]		
	L_{crimp}	L_{exp}	$L_{recoil,exp}$	D_{crimp}	D_{exp}	$D_{recoil,exp}$
Stent V2 01	19.77	19.26	19.04	1.20	3.69	3.65
Stent V2 02	19.98	19.17	19.00	1.20	3.77	3.59
Stent V2 03	19.93	19.26	19.30	1.19	3.63	3.63
Stent V2 04	19.68	18.96	18.83	1.19	3.81	3.77
Stent V2 05	19.52	18.83	18.79	1.20	3.84	3.76
Stent V2 06	19.57	18.96	19.13	1.20	3.75	3.75
Stent V2 07	19.91	19.13	19.17	1.18	3.80	3.76
Stent V2 08	19.85	19.17	19.00	1.19	3.66	3.62
Mean	19.78	19.10	19.02	1.19	3.75	3.70
SD ±	0.16	0.14	0.16	0.01	0.07	0.07

L_{crimp}: stent length after crimping
L_{exp}: stent length at maximum balloon expansion
$L_{recoil,exp}$: stent length after recoil
D_{crimp}: stent diameter after crimping
D_{exp}: stent diameter at maximum balloon expansion
$D_{recoil,exp}$: stent diameter after recoil
