# Supplementary material for: Experimentally validated simulation of coronary stents considering different dogboning ratios and asymmetric stent positioning
Source: PLoS One. 2019 Oct 18;14(10):e0224026. doi: 10.1371/journal.pone.0224026 (PMC6799901; doi:10.1371/journal.pone.0224026)
Supplement: S4 Table — (PDF) [file pone.0224026.s007.pdf]

	Dogboning DB [—]		Stent V2 diameter D_{DB} at Dogboning [mm]		
Stent sample	DB_{prox}	DB_{dist}	$D_{prox,DB}$	$D_{mid,DB}$	$D_{dist,DB}$
Stent V2 01	-0.21	-0.15	2.66	3.35	2.83
Stent V2 02	-0.13	-0.21	2.87	3.30	2.61
Stent V2 03	-0.25	-0.22	2.52	3.37	2.65
Stent V2 04	-0.23	-0.22	2.65	3.42	2.65
Stent V2 05	-0.25	-0.17	2.60	3.46	2.86
Stent V2 06	-0.24	-0.23	2.64	3.50	2.68
Stent V2 07	-0.25	-0.21	2.60	3.46	2.73
Stent V2 08	-0.15	-0.26	3.02	3.54	2.63
Mean	-0.21	-0.21	2.67	3.42	2.71
SD ±	0.05	0.03	0.11	0.07	0.10

Analysis of the dogboning ratio DB based on equation 1

DB_{dist}: dogboning ratio at distal stent end

DB_{prox}: dogboning ratio at proximal stent

D_{DB}: corresponding stent diameter for the analysis of the dogboning ratio
